# Dual role of the miR‐146 family in rhinovirus‐induced airway inflammation and allergic asthma exacerbation

**DOI:** 10.1002/ctm2.427

**Published:** 2021-05-28

**Authors:** Anet Laanesoo, Egon Urgard, Kapilraj Periyasamy, Martti Laan, Yury A. Bochkov, Alar Aab, Nathaniel Magilnick, Margus Pooga, James E. Gern, Sebastian L. Johnston, Jonathan M. Coquet, Mark P. Boldin, Jesper Wengel, Alan Altraja, Grazyna Bochenek, Bogdan Jakiela, Ana Rebane

**Affiliations:** ^1^ Institute of Biomedicine and Translational Medicine University of Tartu Tartu Estonia; ^2^ School of Medicine and Public Health University of Wisconsin‐Madison Madison Wisconsin USA; ^3^ Department of Molecular and Cellular Biology Beckman Research Institute of City of Hope National Medical Center Duarte California USA; ^4^ Institute of Technology University of Tartu Tartu Estonia; ^5^ National Heart and Lung Institute Imperial College London London UK; ^6^ Imperial College Healthcare NHS Trust London UK; ^7^ Department of Microbiology Tumor and Cell Biology (MTC) Karolinska Institutet Stockholm Sweden; ^8^ Nucleic Acid Center Department of Physics Chemistry and Pharmacy University of Southern Denmark Odense Denmark; ^9^ Department of Pulmonary Medicine University of Tartu Tartu Estonia; ^10^ Lung Clinic of the Tartu University Hospital Tartu Estonia; ^11^ Department of Medicine Jagiellonian University Medical College Krakow Poland

**Keywords:** asthma, bronchial epithelial cell, house dust mite, microRNA, neutrophils, noncoding RNA, viral infection

## Abstract

Rhinovirus (RV) infections are associated with asthma exacerbations. MicroRNA‐146a and microRNA‐146b (miR‐146a/b) are anti‐inflammatory miRNAs that suppress signaling through the nuclear factor kappa B (NF‐κB) pathway and inhibit pro‐inflammatory chemokine production in primary human bronchial epithelial cells (HBECs). In the current study, we aimed to explore whether miR‐146a/b could regulate cellular responses to RVs in HBECs and airways during RV‐induced asthma exacerbation. We demonstrated that expression of miR‐146a/b and pro‐inflammatory chemokines was increased in HBECs and mouse airways during RV infection. However, transfection with cell‐penetrating peptide (CPP)‐miR‐146a nanocomplexes before infection with RV significantly reduced the expression of the pro‐inflammatory chemokines CCL5, IL‐8 and CXCL1, increased interferon‐λ production, and attenuated infection with the green fluorescent protein (GFP)‐expressing RV‐A16 in HBECs. Concordantly, compared to *wild‐type* (*wt*) mice, *Mir146a/b^−/−^* mice exhibited more severe airway neutrophilia and increased T helper (Th)1 and Th17 cell infiltration in response to RV‐A1b infection and a stronger Th17 response with a less prominent Th2 response in house dust mite extract (HDM)‐induced allergic airway inflammation and RV‐induced exacerbation models. Interestingly, intranasal administration of CPP‐miR‐146a nanocomplexes reduced HDM‐induced allergic airway inflammation without a significant effect on the Th2/Th1/Th17 balance in *wild*‐*type* mice. In conclusion, the overexpression of miR‐146a has a strong anti‐inflammatory effect on RV infection in HBECs and a mouse model of allergic airway inflammation, while a lack of miR‐146a/b leads to attenuated type 2 cell responses in mouse models of allergic airway inflammation and RV‐induced exacerbation of allergic airway inflammation. Furthermore, our data indicate that the application of CPP‐miR‐146a nanocomplexes has therapeutic potential for targeting airway inflammation.

## Graphical abstract

miR‐146a has anti‐inflammatory properties in human bronchial epithelial cells and mouse airways during rhinovirus infection and in case of allergic inflammation. Particular cell penetrating peptide(CPP)‐miR‐146a nanocomplexes have therapeutic potential for targeting of airway inflammation.

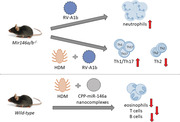

**Abbreviations**
3′UTRs: 3′ untranslated regions
BAL: bronchoalveolar lavage fluid
BMDCs: bone marrow‐derived dendritic cells
CARD10: caspase recruitment domain‐containing protein 10
CCL5: C‐C Motif Chemokine Ligand 5
CPP: cell‐penetrating peptide
CXCL1: chemokine (C‐X‐C motif) ligand 1
ELISA: enzyme‐linked immunosorbent assay
GFP: green fluorescent protein
HBECs: human bronchial epithelial cells
HDM: house dust mite extract
i.n.: intranasal
ICAM‐1: intercellular adhesion molecule 1
IFITM1: interferon‐induced transmembrane protein 1
IFN‐λ: interferon‐λ
ILC2: type 2 innate lymphoid cells
IRAK1: interleukin‐1 receptor‐associated kinase 1
IRF1: interferon regulatory factor 1
LDLR: low‐density lipoprotein receptor
miR‐146a/b: miR‐146a and miR‐146b
miRNA: microRNA
NF‐κB: nuclear factor kappa B
RV: rhinovirus
Th: T helper
Tregs: regulatory T cells
*wt*: *wild*‐*type*

## 1 INTRODUCTION

Asthma is the most common chronic disease of the airways and causes coughing and wheezing due to airway narrowing.^1^ Asthma symptoms are often triggered by various allergic reactions; however, the disease itself is considered very heterogeneous. Accordingly, asthma can be divided into different phenotypes depending on the cell types triggering airway inflammation.^2^, ^3^ The type 2 phenotype includes allergic asthma, which is associated with increased T helper (Th)2 responses and IgE levels, and eosinophilic non‐allergic asthma, which is characterized by type 2 innate lymphoid cells (ILC2) and increased expression of epithelial alarmins.^4^, ^5^ Non‐eosinophilic asthma is characterized by airway neutrophilia and involvement of Th17 cells.^6^, ^7^ To provide asthma control, inhaled corticosteroids and long‐acting bronchodilators are most commonly used.^8^ In addition to allergens, infections with respiratory tract viruses, including human rhinoviruses (RVs), are important triggers for worsening asthma symptoms and may lead to hospitalization or even death.^9^ Among asthma exacerbations caused by viral infections, human RVs make up approximately 65% of cases.^10^

RVs are positive‐strand RNA viruses that infect the upper and lower airways and cause the common cold.^11^, ^12^ RVs can be categorized based on the receptor they use to enter cells.^13^ The majority of RV‐A and all RV‐B serotypes, including RV‐A16, use intercellular adhesion molecule‐1 (ICAM‐1) as their receptor and are classified as major group RVs. The minor group consists of only 10 RV‐A serotypes, which enter cells using the low‐density lipoprotein receptor (LDLR).^14^ From the minor group, RV‐A1b has often been used in research. RV infection and replication in epithelial cells activate innate immune responses and induce expression of growth factors and chemokines, which leads to recruitment and activation of granulocytes, including neutrophils, destruction of normal airway tissue, and development of respiratory symptoms.^13^, ^15^, ^16^

MicroRNAs (miRNAs) are short non‐coding RNAs that modulate various biological processes through posttranscriptional regulation of gene expression.^17^ More specifically, miRNAs recognize and bind to the 3′ untranslated regions (3′UTRs) of multiple target mRNAs via the complementarity of 6‐ to 8‐nt‐long seed regions present in miRNAs. Once mRNA is bound by miRNA, its translation is inhibited or mRNA degradation is triggered by miRNA‐binding proteins.^18^ In this way, miRNAs are also capable of regulating immune responses in various conditions.^19^, ^20^, ^21^ The miR‐146 family consists of miR‐146a and miR‐146b (miR‐146a/b) that differ by only two nucleotides in their 3′ region, which is considered less significant for their interaction with mRNA targets. As such, miR‐146a/b has been reported to have a similar set of target genes.^22^, ^23^, ^24^ Previous studies have demonstrated that multiple components, including interleukin‐1 receptor‐associated kinase 1 (IRAK1) and caspase recruitment domain 10 (CARD10), from the NF‐κB pathway are directly targeted by miR‐146a/b.^23^, ^25^ Accordingly, overexpression of miR‐146a/b has been shown to suppress endogenous levels of IRAK1 and CARD10 and of NF‐κB‐inducible chemokines IL‐8 and chemokine (C‐X‐C motif) ligand 1 (CXCL1) in multiple cell types, including human bronchial epithelial cells (HBECs).^26^ An interesting example is C‐C Motif Chemokine Ligand 5 (CCL5), which has been shown to be directly suppressed by miR‐146a and through IRAK1 and CARD10.^27^ Altered expression of miR‐146a has been identified in various asthma studies,^20^, ^28^, ^29^, ^30^, ^31^ and we previously demonstrated that development of the neutrophilic phenotype of asthma may be among the factors associated with reduced expression of miR‐146a in human airway epithelial cells.^26^ Therefore, we hypothesized that miR‐146a/b may play an important role in the regulation of immune responses to RV infections in bronchial epithelial cells and the airways, as well as in asthma exacerbations.

In this study, we examined the expression and function of miR‐146a/b during RV infection in HBECs and in mouse models of RV‐induced airway inflammation, house dust mite extract (HDM)‐induced allergic asthma, and RV‐induced exacerbation of allergic airway inflammation. We demonstrate that miR‐146a has the capacity to suppress inflammatory responses to RVs in HBECs and during RV‐ and/or HDM‐induced airway inflammation; however, mice lacking miR‐146a/b develop less prominent type 2 cell responses in mouse models of HDM‐induced allergic airway inflammation and RV‐induced exacerbation of allergic airway inflammation.

## 2 RESULTS

### 2.1 MiR‐146a inhibits the expression of pro‐inflammatory chemokines and induces interferon response genes during RV infection in HBECs

**FIGURE 1:**
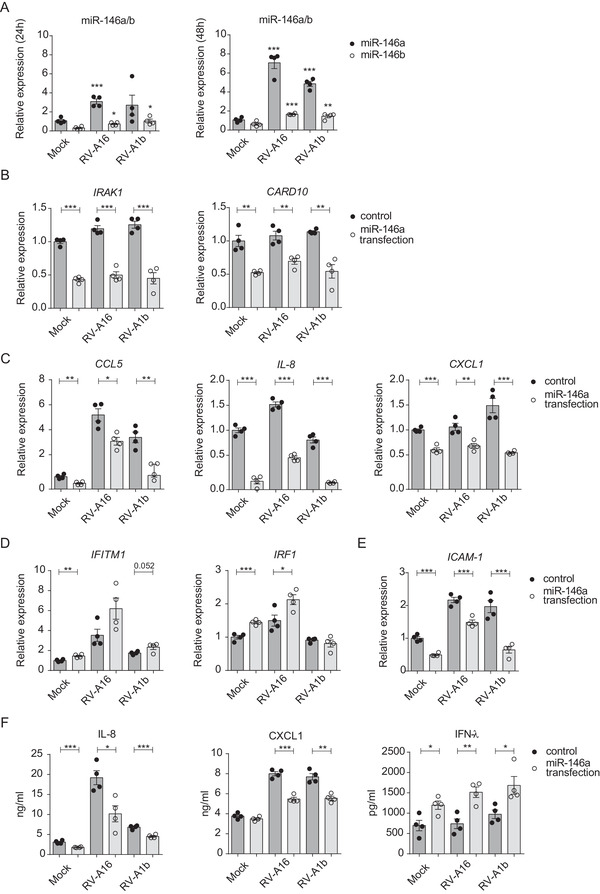
MiR‐146a/b inhibit expression of pro‐inflammatory cytokines and induce interferon response genes in HBECs during RV infection. (A) HBECs were stimulated with RVs for 24 h or 48 h. Relative expression of miR‐146a/b in HBECs was measured by RT‐qPCR and is shown in comparison to corresponding mock‐stimulated cells. (B‐F) HBECs were transfected with miR‐146a or control mimics and stimulated 24 h later with mock or the indicated RVs for 48 h. (B‐E) mRNA expression of the indicated genes in primary HBECs was measured by RT‐qPCR and compared to expression levels of the mock‐stimulated control group mean (= 1). (F) Protein expression of selected genes was measured by ELISA. (A‐F) Data represent mean ± SEM. Unpaired *t*‐test, *p < 0.05, **p < 0.01, ***p < 0.001. One representative of three independent experiments in HBECs from two different donors is shown

To examine the role of miR‐146a/b in the regulation of immune responses to RVs, we first used monolayer cultures of primary HBECs, which is an easy to transfect primary cell culture system that can be infected by RVs. We infected HBECs with RV‐A16 or RV‐A1b and analyzed the expression of miR‐146a/b 24 h and 48 h after infection. A two‐ to six‐fold upregulation of miR‐146a and a two‐ to three‐fold increase in miR‐146b in response to RVs were observed, with higher expression of miR‐146a in all analyzed conditions (Figure 1A). RV infection also altered the expression of miR‐146a target genes from the NF‐κB pathway, including interleukin‐1 receptor‐associated kinase 1 (*IRAK1*) and caspase recruitment domain‐containing protein 10 (*CARD10*), as well as tested pro‐inflammatory chemokines and interferon response genes in HBECs (Figure S1). Since RV infection induced both miR‐146a/b (Figure 1A) and genes that have been shown to be downregulated by miR‐146a, such as *CCL5*,^27^ *CXCL1*, and *IL‐8* ^26^ (Figure S1B and S1C), we hypothesized that endogenous levels of miR‐146a/b are not sufficient to fully suppress these genes and next assessed the influence of transfected miR‐146a mimics during RV infection. As miR‐146a mimics had very similar effects as miR‐146b mimics in keratinocytes,^24^ we used only miR‐146a and control mimics in these experiments (Figure S2). As a result, a significant downregulation in mRNA levels of direct miR‐146a targets, including *IRAK1*, *CARD10, CCL5, IL‐8*, and *CXCL1*, was observed in mock and RV‐infected cells (Figures 1B and 1C) following transfection with miR‐146a. Interestingly, miR‐146a transfection led to increased interferon‐regulated interferon regulatory factor 1 (*IRF1*) and a tendency for enhanced interferon‐induced transmembrane protein 1 (*IFITM1*) during RV‐A16 infection compared to control transfection (Figure 1D). In addition, miR‐146a overexpression significantly reduced the expression of the major group RV receptor *ICAM‐1* (Figure 1E). In line with the mRNA expression results, overexpression of miR‐146a significantly inhibited IL‐8 and CXCL1 protein secretion and simultaneously led to increased interferon‐λ (IFN‐λ) production, as measured by enzyme‐linked immunosorbent assay (ELISA), in the supernatants of miR‐146a‐transfected and RV‐infected HBECs (Figure 1F). To further assess the miR‐146 effect in bronchial epithelial cells during RV infection, we performed pathway analyses of putative miR‐146a/b Targetscan^32^ targets, which expression was shown to be altered during RV infection in HBECs^33^ and determined interferon‐gamma response, TNF‐alpha signaling via NF‐κB, interferon‐alpha response and IL‐2/STAT5 signaling from Molecular Signatures Database (MSigDB) Hallmark Gene Set Collection^34^ as pathways preferentially targeted by miR‐146a/b (Table S1). In summary, these results show that miR‐146a inhibits the expression of NF‐κB‐dependent pro‐inflammatory genes while stimulating interferon response genes during RV infection in HBECs.

### 2.2 MiR‐146a reduces the number of infected cells and inhibits neutrophil migration during RV infection in HBECs

**FIGURE 2:**
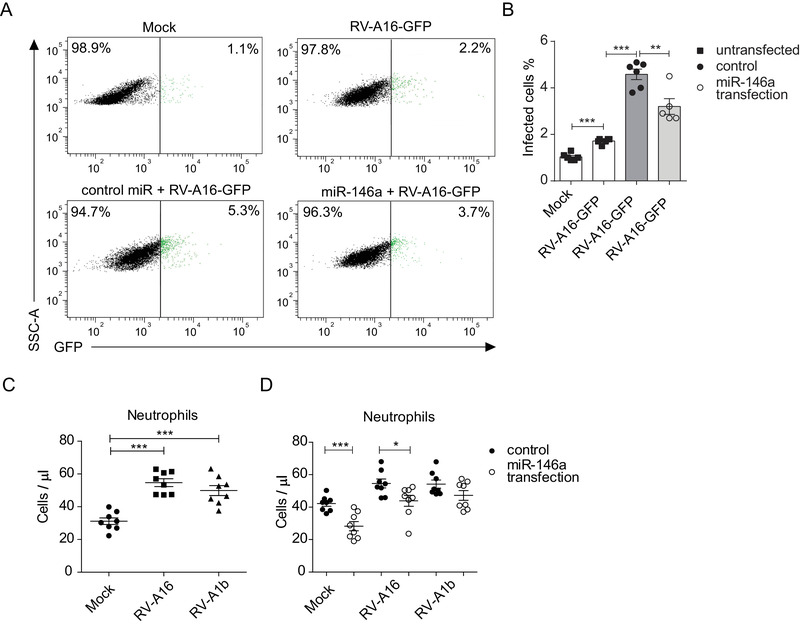
MiR‐146a inhibits RV infection and neutrophil migration. HBECs were transfected with miRNA mimics for 24 h and stimulated with the indicated RVs for 48 h or stimulated with only RV or mock. (A) Representative flow cytometry plots of HBECs infected with RV‐A16‐GFP (green) and (B) corresponding quantification graph. (C‐D) Chemotaxis assay of human neutrophils toward supernatants of primary HBECs stimulated with RVs (C) or toward supernatants of HBECs transfected with the indicated miRNA mimics and stimulated with RVs (D). (C‐D) Data from two independent experiments in HBECs from two different donors are shown. Data represent mean ± SEM. Unpaired *t*‐test, *p < 0.05, **p < 0.01, ***p < 0.001

Because miR‐146a overexpression induced *IRF1* and IFN‐λ expression and inhibited RV‐A16 receptor *ICAM‐1* mRNA levels (Figures 1D‐1F), we next assessed whether miR‐146a influences RV infection in HBECs. HBECs were transfected with miR‐146a mimics, infected with RV‐A16‐green fluorescent protein (GFP), and then assessed by fluorescence microscopy (Figure S3B) or flow cytometry (Figures 2A and 2B). In HBECs transfected with miR‐146a mimics, significantly fewer GFP‐positive cells (3.7% ± 0.2%) were detected than in control cells (5.3% ± 0.3%) (Figures 2A and 2B). The percentage of RV‐infected HBECs was in line with previous studies indicating that only a fraction of HBECs is infected by RVs.^10^ However, transfection nonspecifically enhanced the infection of HBECs with RV‐A16‐GFP. Similar to *wild*‐*type* RV‐A16, RV‐A16‐GFP had the capacity to stimulate miR‐146a/b and *IL‐8, CXCL1*, and *ICAM‐1* mRNA expression in HBECs (Figure S3A, S3C and S3D).

As RV infection in HBECs induced neutrophil‐attracting chemokines, *IL‐8* and *CXCL1*, and miR‐146a transfection inhibited secretion of these chemokines (Figures 1C, 1F, and S1B), we next performed a neutrophil chemotaxis assay. Indeed, increased neutrophil migration toward the supernatants of RV‐A16‐ or A1b‐infected HBECs was detected compared to mock‐infected HBECs (Figure 2C), and significantly fewer neutrophils migrated toward the supernatants of miR‐146a mimic‐transfected and RV‐A16‐infected HBECs compared to the corresponding control. There was a similar tendency with RV‐A1b as well (Figure 2D). In summary, the overexpression of miR‐146a reduces the number of infected HBECs and inhibits the secretion of neutrophil chemoattractants from HBECs during RV infection.

### 2.3 *Mir146a/b^−/−^* mice develop more severe airway neutrophilia in a mouse model of RV‐induced airway inflammation

**FIGURE 3:**
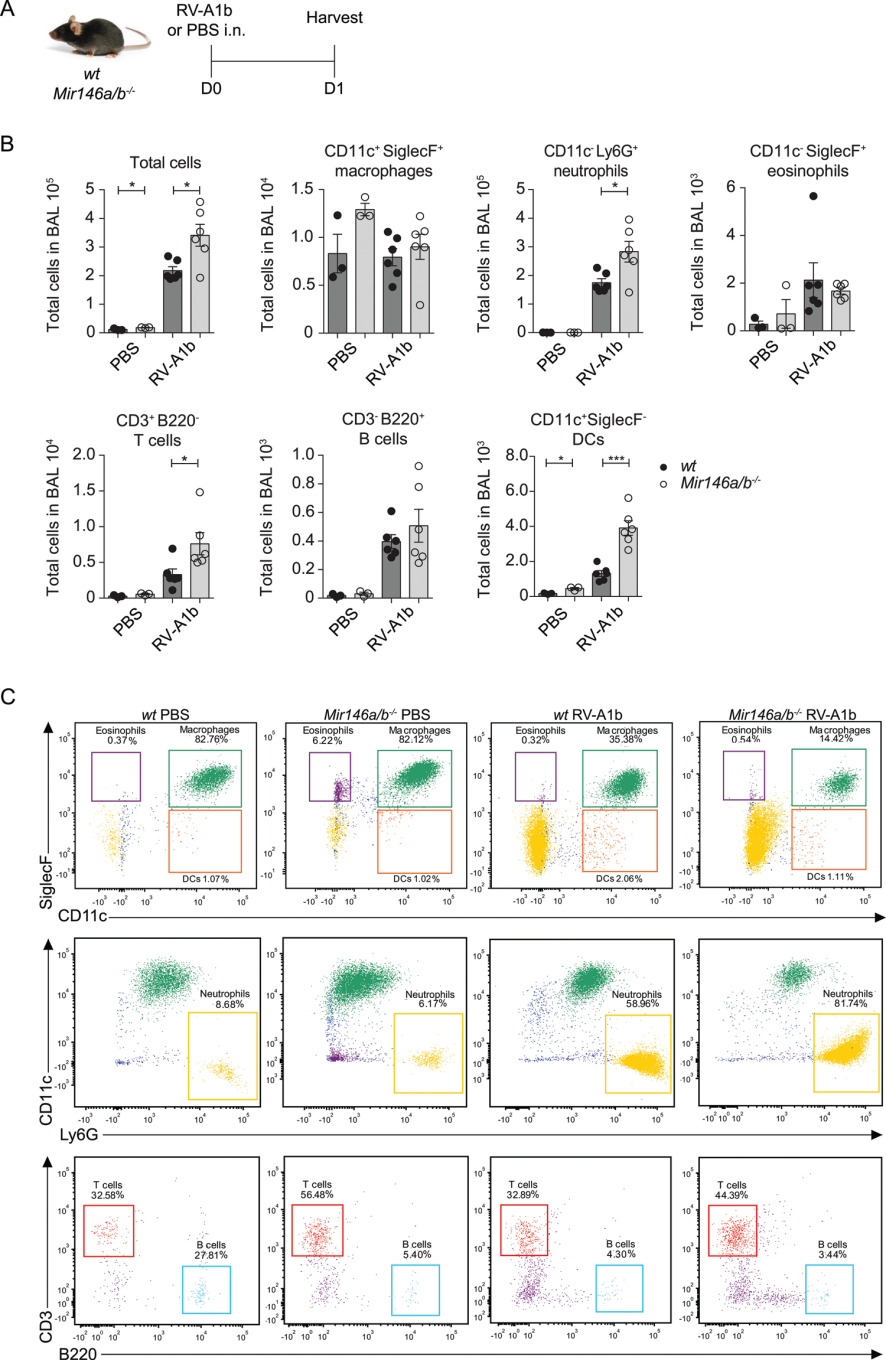
*Mir146a/b^−^^/^^−^* mice exhibit increased airway neutrophilia in a mouse model of RV‐induced airway inflammation. (A) RV‐A1b or PBS was administered intranasally (i.n.) to *wt* or *Mir146a/b^−/−^* mice. Twenty‐four hours after RV‐A1b infection, BAL was analyzed. (B‐C) BAL cells were counted using a hemocytometer and then subjected to FACS analysis, and according to dot plots, the total numbers of immune cells were calculated. Data represent mean ± SEM. Unpaired *t*‐test, *p < 0.05, ***p < 0.001. (C) One representative FACS dot plot of BAL fluid from three PBS‐treated and five RV‐A1b‐treated *wt* and *Mir146a/b^−/−^* mice. Eosinophils, macrophages, DCs, and neutrophils (upper and middle panel) were analyzed as a percentage of the granulocyte population, and T cells and B cells (bottom panel) were analyzed as a percentage of the lymphocyte population

**FIGURE 4:**
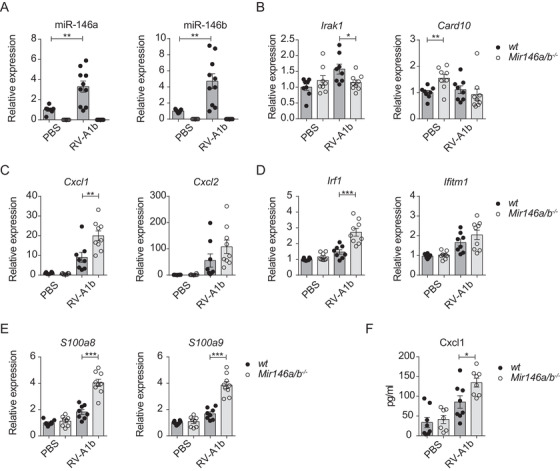
Gene expression of *wt* and *Mir146a/b^−^^/^^−^* mouse lungs in an RV‐induced mouse model of airway inflammation. Twenty‐four hours after i.n. administration of PBS or RV‐A1b, mouse lung lobes were collected. (A) Relative miRNA expression and (B‐E) mRNA expression in lung lobes was measured with RT‐qPCR and are shown compared to the PBS *wt* group mean (= 1). (F) Protein content in BAL measured with ELISA. Data from two independent experiments. Data represent mean ± SEM. Unpaired *t*‐test, *p < 0.05, **p < 0.01, ***p < 0.001

To investigate whether miR‐146a/b may influence immune responses to RVs *in vivo*, a previously developed model that uses the capacity of minor group RVs to infect mouse airways, was applied.^35^ Accordingly, RV‐A1b or PBS was applied intranasally (i.n.) to C57Bl/6J *Mir146a/b^−/−^* and *wild‐type* (*wt*) mice. Twenty‐four hours later, mice were sacrificed, and bronchoalveolar lavage fluid (BAL) and lung tissue were collected (Figure 3A). First, total BAL cells and the number of neutrophils and lymphocytes were significantly elevated in *Mir146a/b^−/−^* mice in response to RV‐A1b infection, as observed when BAL cells were differentially stained and counted by microscopy (Figure S4). Flow cytometry analysis (for sorting strategy, please see Figure S5A) revealed similarly strong increases in total cell number, neutrophils, T cells, B cells and dendritic cells (DCs) in BAL collected from mice infected with RV‐A1b, with further increases in BAL total cell count, neutrophils, T cells, and DCs in *Mir146a/b^−/−^* mice (Figures 3B and 3C). No significant differences in the numbers of B cells, macrophages, or eosinophils were found between *wt* and *Mir146a/b^−/‐^* mice in response to RV infection (Figures 3B and 3C). In addition to BAL cells, we analyzed the expression of miR‐146a/b and relevant genes in lung tissue. Similar to *in vitro* results in HBECs (Figure 1A), RV‐A1b infection stimulated the expression of miR‐146a/b in *wt* mouse lungs (Figure 4A), while no miR‐146a/b was detected in *Mir146a/b^−/−^* mice. Analysis of mRNA levels revealed a significant decrease in expression of the miR‐146a/b direct target gene *Irak1* during RV‐A1b infection and increased *Card10* expression in the PBS treatment group in *Mir146a/b^−/−^* mouse lungs compared to *wt* mice (Figure 4B). Although *Card10* and *Irak1* mRNA was not upregulated in infected *Mir146a/b^−/−^* mice, RV infection more strongly induced *Cxcl1* mRNA expression in the lungs (Figure 4C) and protein levels in the BAL fluid (Figure 4F) in *Mir146a/b^−/−^* mice than in *wt* mice. In addition, we observed significantly increased expression of *Irf1* (Figure 4D) and neutrophil markers *S100a8* and *S100a9* (Figure 4E) in the lungs of *Mir146a/b^−/−^* mice compared to *wt* mice in response to RV infection. Differences in expression levels of *Cxcl2* (Figure 4C) and *Ifitm1* (Figure 4D) were not statistically significant between RV‐A1b‐stimulated *Mir146a/b^−/−^* and *wt* mouse lungs. Taken together, these findings reveal that the lack of miR‐146a/b in mice leads to increased numbers of neutrophils, DCs, and T cells in the airways with concomitantly enhanced expression of the neutrophil chemoattractant *Cxcl1*, as well as neutrophil markers *S100a8* and *S100a9* in the lung tissue during RV‐A1b infection.

### 2.4 *Mir146a/b^−/−^* mice exhibit reduced Th2 cell responses in the airways in models of HDM‐induced allergic airway inflammation and RV‐induced exacerbation of allergic airway inflammation

**FIGURE 5:**
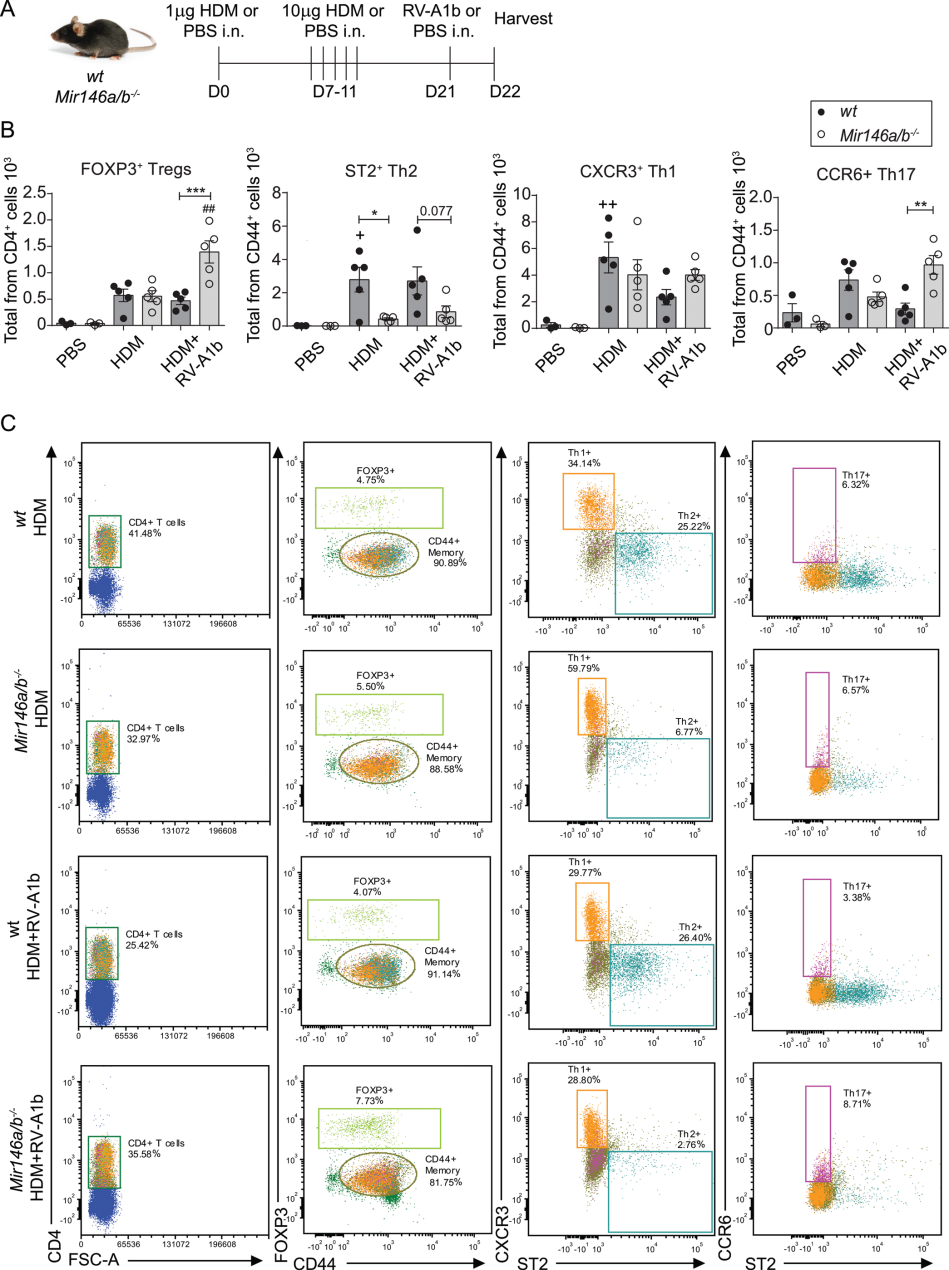
Lack of miR‐146a/b in mice results in an altered immune response in allergic airway inflammation and RV‐induced exacerbation of allergic airway inflammation models. (A) Mice were sensitized (D0) and challenged (D7‐11) with house dust mite extract (HDM) and, when indicated, infected with RV‐A1b (D21) for 24 h. Control mice received only PBS. (B) BAL cells were counted with a hemocytometer and then subjected to FACS analysis. From the dot plot percentages, the total numbers of immune cells were calculated. Data represent mean ± SEM. One‐way ANOVA with Tukey's multiple comparisons test and adjusted *p*‐values are shown. + *p* < 0.05, ++ *p* < 0.01 HDM compared to the same mouse line with PBS; ## *p* < 0.01 HDM+RV‐A1b compared to same mouse line with HDM; *p < 0.05, **p < 0.01, ***p < 0.001 *wt* compared to *Mir146a/b^−/−^* mice for the same treatment. (C) One representative FACS dot plot of BAL fluid from five HDM‐treated or HDM+RV‐A1b‐treated *wt* and *Mir146a/b^−/−^* mice. Data from one representative of three independent experiments are shown

**FIGURE 6:**
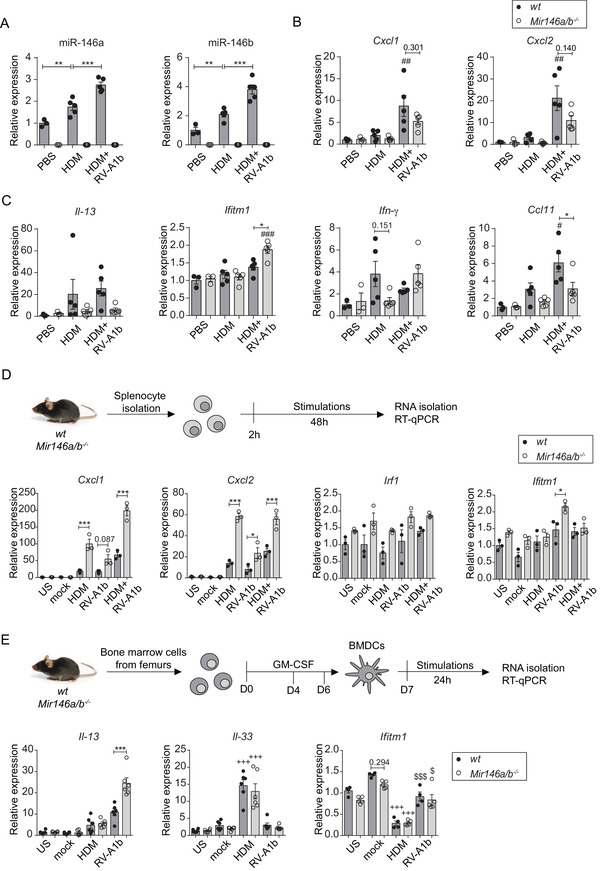
*Mir146a/b^−^^/^^−^* mice display differential gene expression in their lungs, splenocytes, and DCs in response to HDM or RV‐A1b. Relative miRNA (A) and mRNA expression in (B‐C) mouse lungs subjected to HDM‐induced airway inflammation and RV‐induced exacerbation of allergic airway inflammation models, (D) in splenocytes and (E) in BMDCs as measured by RT‐qPCR. Data are presented as the mean ± SEM and are compared to mean values of the *wt* PBS group (= 1) using one‐way ANOVA with Tukey's multiple comparisons test. Adjusted *p*‐values are shown. (A) **p < 0.01 *wt* PBS compared to *wt* HDM, ***p < 0.001 *wt* HDM compared to *wt* HDM+RV‐A1b. Data from one representative of three independent experiments are shown. (B‐C) #*p* < 0.05, ##0.01, ###*p* < 0.001 HDM+RV‐A1b compared to the same HDM mouse line; *p < 0.05 *wt* compared to *Mir146a/b^−/−^* mice during the same treatment. (D) Splenocytes from *wt* and *Mir146a/b^−/−^* mice were stimulated for 48 h; *p < 0.05, **p < 0.01, ***p < 0.001 *wt* compared to *Mir146a/b^−/−^* mice during the same treatment. (E) BMDCs from *wt* and *Mir146a/b^−/−^* mice were stimulated for 24 h; $ *p* < 0.05, $$$ *p* < 0.001 RV‐A1b compared to the same line mock; +++ *p* < 0.001 HDM compared to the same line US; ***p < 0.001 *wt* compared to *Mir146a/b^−/−^* during same treatment

We next studied the susceptibility of mice lacking miR‐146a/b to allergic airway inflammation and RV‐induced exacerbation of allergic airway inflammation. To that end, we used a previously developed mouse model involving repeated i.n. application of (HDM) extract^36^, ^37^ and additionally applied RV‐A1b i.n. to induce exacerbation^35^ in some mice (Figure 5A). Flow cytometry analysis of BAL cells (for sorting strategy, please see Figure S5B) revealed an increased total number of cells, T cell subsets, and B cells, as well as a tendency for enhanced levels of macrophages and eosinophils in response to HDM treatment in *wt* and *Mir146a/b^−/−^* mice when compared to the PBS control group, with a robust increase in neutrophils when mice were additionally subjected to RV infection (Figures S6 and S7). When HDM was applied alone, significantly increased numbers of B cells and strongly reduced numbers of Th2 cells (Figures 5B, 5C, S6, and S7A) were observed in BAL fluid of miR‐146a/b‐deficient mice compared to *wt* mice. When RV was additionally applied, significantly elevated FOXP3^+^ regulatory T cells (Tregs) and a tendency for an increased number of CD44^+^ memory T cells were found in the BAL fluid of *Mir146a/b^−/−^* mice compared to *wt* mice (Figures 5B, 5C, and S7). In the same model, flow cytometry analysis of T helper cell subsets revealed significantly more Th17 cells and a trend for increased numbers of Th1 cells in *Mir146a/b^−/‐^* mice, whereas *wt* mice displayed a tendency toward a higher number of Th2 cells in BAL fluid (Figures 5B‐5C, and Figure S7). Further RT‐qPCR analysis revealed increased miR‐146a/b expression in *wt* mouse lungs in response to HDM treatment, which was further elevated following RV infection (Figure 6A). In line with flow cytometry results, HDM treatment followed by RV infection resulted in increased expression of *Cxcl1, Cxcl2*, and *Ccl11* in *wt* mouse lungs compared to mice treated with HDM alone, confirming the exacerbation of inflammation in the lungs (Figures 6B and 6C). When *wt* and *Mir146a/b^−/‐^* mice were compared, a tendency for decreased *Cxcl1, Cxcl2, Il‐13, Il‐4, Il‐17a, S100a8, S100a9* expression (Figures 6B, 6C, and S8A) and significantly decreased *Ccl11* and increased *Ifitm1* expression (Figure 6B‐C) were detected in lungs from *Mir146a/b^−/‐^* mice following HDM and RV‐A1b application. Differences in the expression levels of *Irf1* and *Ifn‐γ* were not statistically significant between *wt* and *Mir146a/b^−/−^* mice.

To further compare the responses of the immune system between these two mouse lines, we extracted splenocytes and bone marrow‐derived DCs (BMDCs) and stimulated these cells *in vitro* using HDM or RV‐A1b alone or in combination. In contrast to the *in vivo* HDM‐induced airway inflammation and RV‐induced exacerbation of allergic airway inflammation models (Figure 6B), however consistent with the RV‐induced airway inflammation model (Figure 4C), a significantly increased expression of *Cxcl1* and *Cxcl2* was detected in *Mir146a/b^−/‐^* mouse splenocytes (Figure 6D) in response to HDM alone but also in the case of HDM and RV‐A1b co‐stimulation. *Mir146a/b^−/‐^* mouse splenocytes exhibited elevated *Ifitm1* expression following the addition of RV‐A1b, while no significant changes in *Irf1* were observed (Figure 6D). In addition, we detected elevated expression of *Il‐33* and decreased expression of *Ifitm1* in bone marrow‐derived DCs (BMDCs) from both mouse lines, with no significant differences between *Mir146a/b^−/‐^* and *wt* mice in response to HDM stimulation, while significantly higher expression of *Il‐13* was observed in RV‐A1b stimulated *Mir146a/b^−/‐^* BMDCs compared to cells from *wt* mice (Figure 6E). No differences in expression levels of *Cxcl1, Cxcl2*, or *Irf1* were found between *Mir146a/b^−/‐^* and *wt* BMDCs (Figure S8B). Together, these results demonstrate that *Mir146a/b^−/‐^* mice develop more dominant Th1/Th17 responses and less prominent Th2 cell responses in mouse models of HDM‐induced allergic airway inflammation and RV‐induced exacerbation of allergic airway inflammation, while BMDCs lacking miR‐146a/b did not show a gene expression pattern supporting Th1/Th17 skewing.

### 2.5 Intranasal administration of CPP‐miR‐146a nanocomplexes reduces HDM‐induced allergic airway inflammation

**FIGURE 7:**
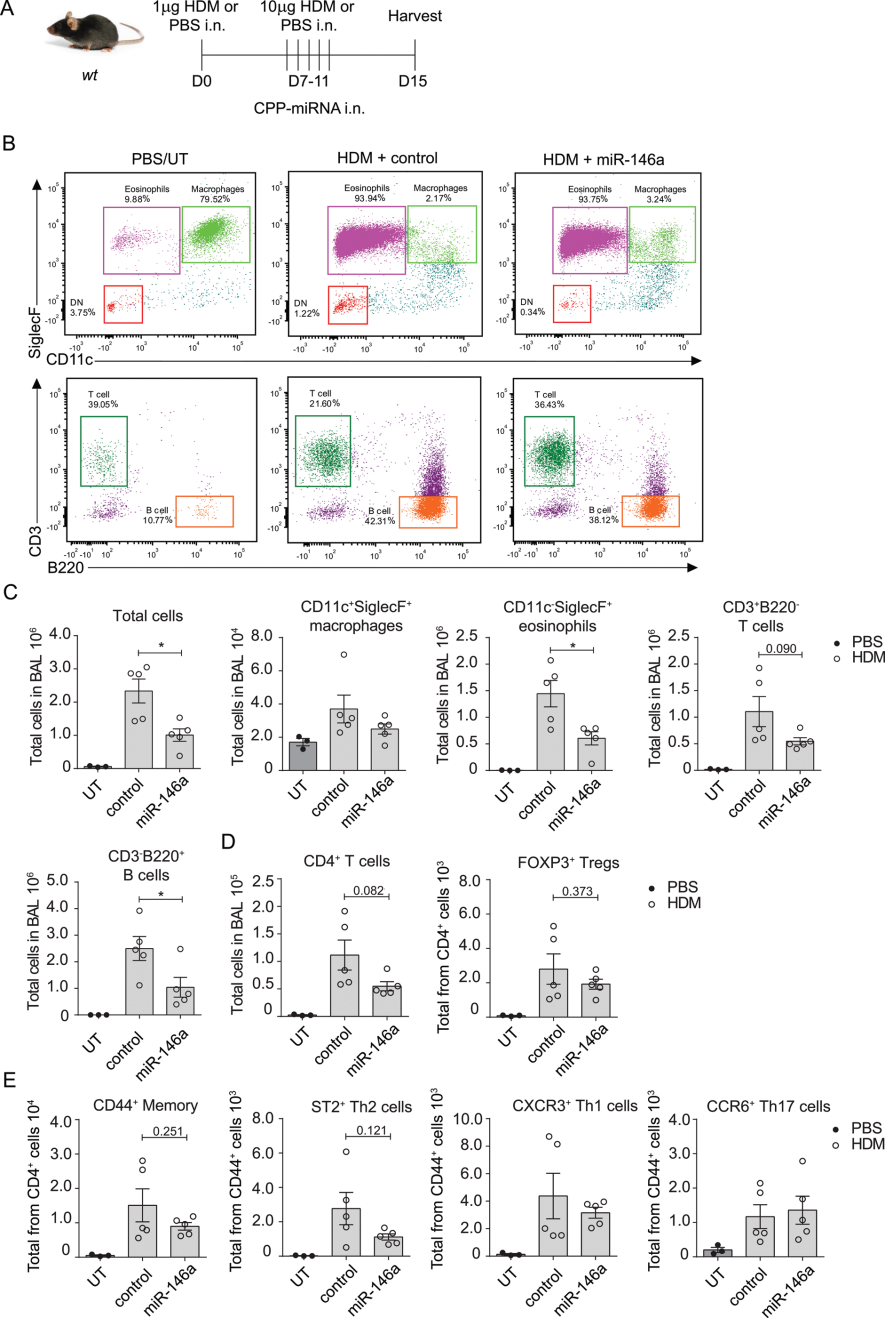
Administration of CPP‐miR‐146a nanocomplexes inhibits the accumulation of eosinophils, T cells, and B cells in an allergic airway inflammation model. Wt mice were sensitized i.n. at day 0 with 1 μg and challenged at days 7–11 daily with 10 μg of HDM. CPP‐miR‐146a (miR‐146a) and CPP‐miRNA control (control) nanocomplexes containing 60 pmol of corresponding miRNA mimic were applied 2 h after each challenge. On day 15, BAL cells and lung lobes for RNA were harvested. PBS group was left untransfected (UT). (A) Schematic of the experimental setup. (B and C) BAL fluid cells were counted with a hemocytometer, then subjected to FACS analysis. From the dot plots, the total numbers of immune cells were calculated. (B) One representative FACS dot‐plot of BAL macrophages, eosinophils, CD3+ T cells, and B220+ B cells. (C) Data are represented as mean ± SEM from five mice in study groups and three mice in UT PBS group, unpaired *t*‐test, *p < 0.05. Data from one representative of two independent experiments are shown. (C‐E) BAL cells were counted using a hemocytometer and then subjected to FACS analysis. From the dot plots, the total numbers of immune cells were calculated. Data are represented as the mean ± SEM, unpaired *t*‐test, *p < 0.05. Data from one representative of two independent experiments are shown

**FIGURE 8:**
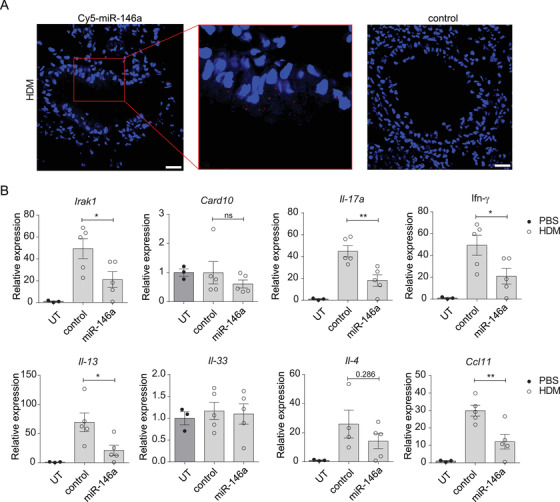
CPP‐miR‐146a nanocomplexes inhibit the expression of pro‐inflammatory genes in an HDM‐induced airway inflammation model. (A) Localization of labeled Cy5‐miR‐146a mimics (red) in lung lobes counterstained with DAPI (blue). Scale bar = 100 μm. (B) Relative mRNA expression levels of the indicated genes were compared to the PBS‐treated untransfected (UT) group (= 1) or HDM‐treated and miR‐146a mimic‐ or control‐transfected mouse lung lobes measured by RT‐qPCR. Data are represented as the mean ± SEM. Unpaired *t*‐test, *p < 0.05, **p < 0.01, ***p < 0.001. Data from one representative of two independent experiments are shown

The transfection of miR‐146a mimics suppressed RV‐induced inflammation in HBECs (Figure 1, 2), and *Mir146a/b^−/−^* mice developed increased inflammation in the RV infection model (Figure 4). However, *knockout* mice displayed less prominent Th2 cell responses in HDM‐induced allergic airway inflammation and RV‐induced exacerbation of allergic airway inflammation models (Figures 5 and 6). Therefore, we next assessed whether *in vivo* application of miR‐146a has therapeutic effects in the case of allergic airway inflammation in mice. We subjected wt mice to an HDM‐induced allergic inflammation model (54, 55) and administered CPP‐miR‐146a or control nanocomplexes either 2 h before (Figure S9) or after each challenge (Figure 7A), followed by flow cytometry analysis of BAL fluid cells and RT‐qPCR of total RNA from the lung. When nanocomplexes were applied 2 h before the challenge, the treatment with HDM resulted in increase in numbers of measured cell counts without any significant differences between the HDM‐treated groups. Note that cell counts in HDM‐treated groups did not depend on whether CPP‐miRNAs were included, indicating that application of CPP‐miRNA nanocomplexes does not increase airway inflammation (Figure S9). A significantly reduced total number of eosinophils and B cells were found in BAL following administration of CPP‐miR‐146a nanocomplexes compared to CPP‐control from HDM‐treated mice (Figures 7B, 7C, S10A). A tendency for reduced numbers of regulatory T cells, memory T cells, and Th2 cells was found; however the differences were statistically not significant (Figures 7D and 7E). We also evaluated the delivery efficiency and localization of administered CPP‐miRNA nanocomplexes in mouse airways using Cy5‐labeled miR‐146a mimics and confocal microscopy. Figure 8A demonstrates that Cy5‐miR‐146a mimics localize around bronchioles in frozen mouse lung lobe sections. No Cy5 signal was detected in the kidney, liver, spleen, or MLN sections from mice transfected with CPP‐miR‐146a nanocomplexes when analyzed by confocal microscopy (data not shown). In line with this, RT‐qPCR analysis of total lung tissue RNA demonstrated that administration of CPP‐miR‐146a nanocomplexes significantly inhibited HDM‐induced expression of *Il‐13, Il‐17A, Ifn‐γ*, and the eosinophil attracting chemokine *Ccl11* (Figure 8B). In addition, a tendency for decreased expression of *Cxcl1, Cxcl2, Irf1*, and *Ifitm1* was observed (Figure S10B) in mouse lungs in the presence of CPP‐miR‐146a nanocomplexes. Taken together, our results demonstrate that the application of CPP‐miR‐146a nanocomplexes exerts an anti‐inflammatory effect in mouse airways during HDM‐induced allergic inflammation.

**FIGURE 9:**
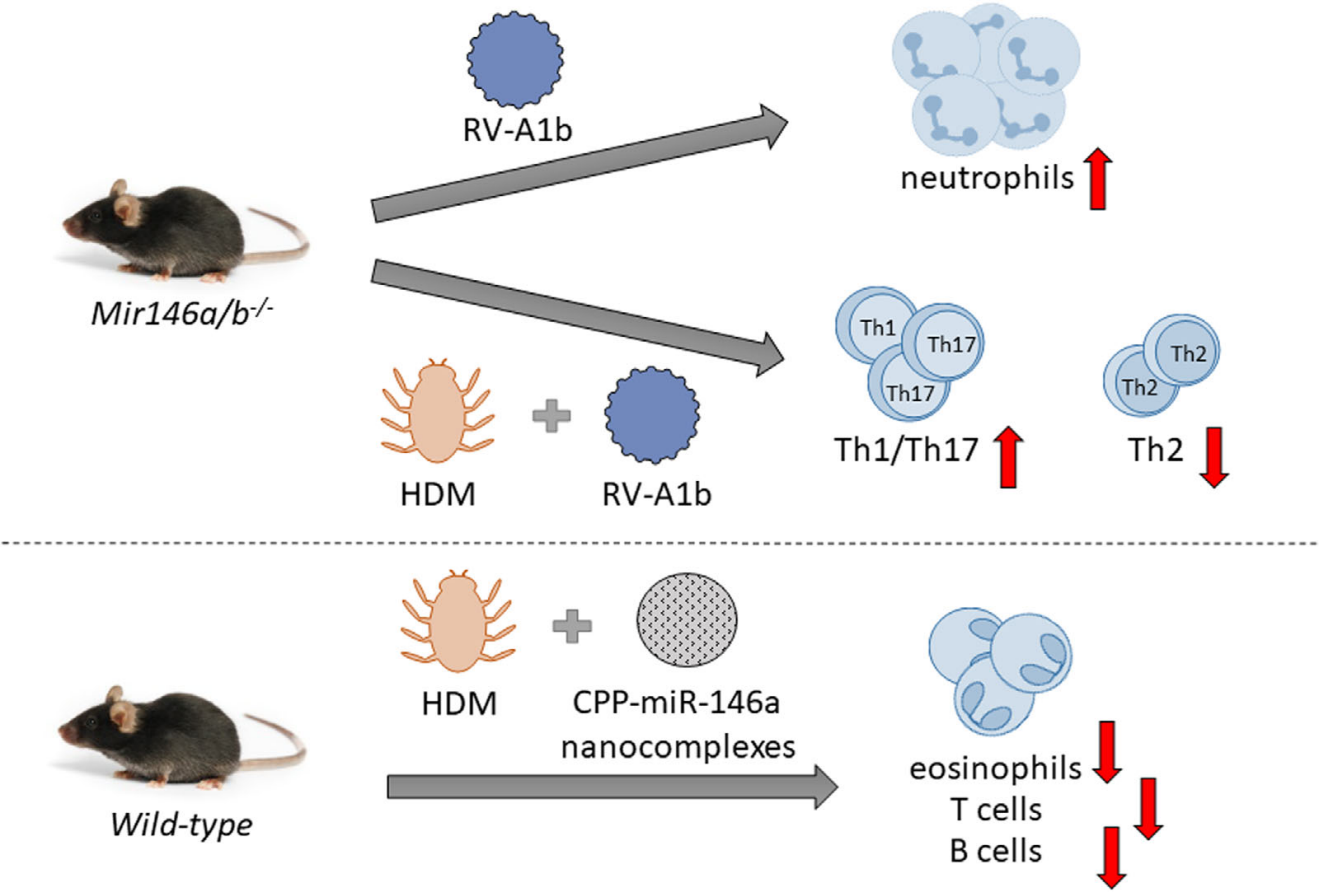
miR‐146a/b‐deficient mice develop more severe airway neutrophilia during RV infection and a less prominent Th2 cell mediated immune response in HDM‐induced allergic airway inflammation and RV‐induced exacerbation of allergic airway inflammation when compared with *wild‐type* mice. Application of cell‐penetrating peptide (CPP)‐miR‐146a nanocomplexes into the airways has an anti‐inflammatory effect in the HDM‐induced allergic airway inflammation model in *wild‐type* mice.

## 3 DISCUSSION

Numerous studies have reported altered miRNA expression in asthmatic patients,^38^, ^39^, ^40^, ^41^ and accumulating data on miRNA functions from cell culture experiments^42^ and animal models^43^ suggest that miRNAs play important roles in the regulation of airway inflammation and the development of asthma. In the current study, we used both cell culture and mouse models to delineate miR‐146a/b functions in airway inflammation. We demonstrated that miR‐146a inhibits the pro‐inflammatory chemokines IL‐8, CXCL1, and CCL5, induces the production of IFN‐λ, and has the capacity to limit infection by RV‐A16 in HBECs. In line with the results from cell culture experiments, mice lacking miR‐146a/b displayed increased neutrophil infiltration and elevated secretion of chemokines in an RV‐induced airway inflammation model. Interestingly, while *Mir146a/b^−/−^* mice had less prominent Th2 responses in airway inflammation models involving HDM as an allergen, the application of CPP‐miR‐146a nanocomplexes led to general anti‐inflammatory effects in allergic airway inflammation, suggesting therapeutic potential for these nanocomplexes (Figure 9).

The first set of experiments demonstrated that miR‐146a/b expression is induced in response to RV‐A16 and RV‐A1b in HBECs along with changes in the studied pro‐inflammatory chemokines and interferon response genes. Previous publications have shown that miR‐146a and miR‐146b expression is dependent on activation of the NF‐κB and JAK‐STAT pathways, respectively,^23^, ^44^, ^45^ which is in line with studies showing that both of these signaling pathways are induced during RV‐induced inflammation in the airways.^46^, ^47^, ^48^, ^49^

When miR‐146a was transfected into HBECs, we observed significantly reduced expression of neutrophil attracting chemokines, IL‐8 and CXCL1,^50^, ^51^ and fewer migrating neutrophils toward supernatants of RV‐stimulated HBECs. This finding suggests that miR‐146a may act to relieve RV‐induced inflammation mediated by neutrophils and is in line with our previous results demonstrating a link between decreased expression of miR‐146a in airway epithelial cells and the development of a neutrophilic phenotype of asthma.^26^

Interestingly, we observed elevated levels of IFN‐λ in the supernatants and reduced mRNA levels of the RV‐A16 receptor ICAM‐1 in HBECs transfected with miR‐146a and infected with RVs. Moreover, a reduced infection rate of HBECs transfected with miR‐146a and infected with GFP‐tagged RV‐A16 was detected. It has been previously shown that both type I and type III interferon responses are activated during RV infection and that this activation is needed to limit viral spread.^47^, ^48^ Thus, our results indicate that miR‐146a may limit the viral spread of RVs through its capacity to increase IFN‐λ and additionally may inhibit the major group of RVs through suppression of ICAM‐1. Similar antiviral properties of miR‐146a in A549^52^ and other bronchial epithelial cell lines^42^, ^53^ have been reported, however, not in primary epithelial cells.

To assess the function of the miR‐146 family during RV infection *in vivo*, we next performed a set of experiments using *wt* and miR‐146a/b *knockout* mice. We opted to use *Mir146a/b^−/−^* mice, as miR‐146a/b has identical seed sequences, can affect the same target genes^44^, ^45^ and therefore may partially compensate for each other.^54^, ^55^ We first generated a short mouse model of RV‐induced airway inflammation, which involved infection of mice with RV‐A1b. As major group RVs do not bind to mouse *Icam‐1*,^56^, ^57^ we only used RV‐A1b in this experiment. We found an increased total number of cells in the airways, including neutrophils, DCs, and T cells, as well as enhanced expression of *Cxcl1, S100a8, and S100a9* after RV‐A1b infection in *Mir146a/b^−/‐^* mice compared to *wt* mice. Interestingly, there was no difference in *Ifitm1* mRNA levels, and *Irf1* was even increased in *Mir146a/b^−/^* ^−^ mice infected with RV compared to the control group, while *in vitro* results in HBECs showed that transfection of miR‐146a resulted in increased expression of interferon response genes. This difference might be due to the effect of other cell types present in the airways. Previously, the involvement of *Irf1* in the regulation of type III interferons and its capacity to control the spread of influenza A virus have been shown.^58^, ^59^ In our experiments, the higher airway expression of *Irf1* in RV‐infected mice lacking miR‐146a/b was accompanied by a greater pro‐inflammatory response. As suggested by previous publications, airway neutrophilia^13^ and T‐cell recruitment^60^, ^61^ act to mediate viral clearance via Th1 cytokine production during RV infection. However, if cellular and immune responses are too strong during viral infection, a more serious health condition may develop. Our data suggest that the miR‐146 family is a part of an immune regulatory network needed for optimal immune responses in the airways during RV infection. Accordingly, we next studied how the lack of the miR‐146 family influences immune responses in RV‐induced exacerbation of allergic airway inflammation. As asthma exacerbations occur seasonally peaking from fall to winter, it has been suggested that despite the phase of an allergic reaction, viral infections play a major role in the exacerbation of asthma.^62^ For example, it has been shown that RV infection occurring even 1 month after antigen exposure increases the occurrence of a late‐phase asthmatic response to the allergen by causing prolonged eosinophil infiltration to the lower airways of patients.^63^ Therefore, we modified the previous protocol according to which mice are infected together with the last challenge^35^ and inoculated mice with RV A1b 10 days after the last challenge. Surprisingly, in this model, flow cytometry analysis revealed no significant differences in the number of total cells in BAL fluid after HDM and RV co‐administration between the two mouse lines. However, strongly reduced numbers of Th2 cells were detected after HDM treatment, while significantly increased Th17 and FOXP3+ regulatory T cell infiltrations were found after HDM and RV application to the airways of *Mir146a/b^−/−^* mice. Similarly, we observed significantly decreased expression of eosinophils attracting chemokine *Ccl11* and elevated expression of *Ifitm1* in the lungs of *Mir146a/b^−/−^* mice following HDM and RV‐A1b administration. Previously, a loss of peripheral T cell tolerance, excessive amounts of Ifn‐γ expressed by CD4^+^ T cells, and elevated Th17 numbers have been reported for miR‐146a‐deficient mice due to the overactivation of STAT1 when mice are 6–8 months old.^54^, ^64^, ^65^, ^66^, ^67^, ^68^ Our results indicate that Th2 responses may already be impaired in *Mir146a/b^−/‐^* mice at 8–10 weeks old or similarly to *Mir146a^−/−^* mice, dampened due to excessive Th17/Th1 cell‐mediated immune responses revealing in inflammatory conditions. In line with results from the RV‐induced airway inflammation model, increased mRNA expression of *Cxcl1, Cxcl2*, and *Ifitm1* in HDM‐stimulated *Mir146a/b^−/−^* splenocytes was detected. However, the mRNA levels of other cytokines, such as *Il‐17A, Il‐13, Il‐33*, and *Il‐4*, were very low under these conditions (data not shown). As BMDCs from *Mir146a/b^−/−^* mice expressed increased *Il‐13*, the dominance of Th17/Th1 cell‐mediated immune responses in *Mir146a/b^−/−^* mice is likely not caused by differences in antigen‐presenting cells. As one possible scenario, T helper cells from *Mir146a/b^−/−^* mice might be more prone to Th17/Th1 type cytokine expression when activated, and that further suppresses the development of Th2 responses, eosinophilia, and inflammation during HDM treatment. Also, differences in epithelial or other tissue‐resident cell responses may lead to impaired Th2 responses in *Mir146a/b^−/−^* mice. Therefore, the precise mechanism that leads to reduced Th2 responses at the organism level in the absence of miR‐146a/b remains to be studied.

Since *Mir146a/b^−/−^* mice reacted somewhat differently in response to RV‐induced inflammation and exacerbation of allergic airway inflammation, we next assessed the effect of CPP‐miR‐146a nanocomplexes in a mouse model of HDM‐induced allergic airway inflammation, a Th2‐dependent model with a high number of eosinophils.^69^ Confocal microscopy of mouse lungs revealed efficient delivery of Cy5‐labeled miR‐146a mimics into airway cells. Interestingly, *wt* mice receiving CPP‐miR‐146a nanocomplexes displayed a significantly reduced total number of cells, eosinophils, and B cells in their BAL following HDM administration. In addition, decreased expression of *Il‐17a, Ifn‐γ, Il‐13*, and *Ccl11* upon HDM exposure was found in lung lobes in mice treated with CPP‐miR‐146a. Taken together, these results suggest that the administration of CPP‐miR‐146a nanocomplexes affects all immune responses, leading to decreased inflammation and attenuated airway symptoms without significant changes in the Th2/Th1/Th17 balance.

Generally, the therapeutic application of miRNA mimics is limited due to their low capacity to penetrate cell membranes due to their negative charge.^70^ To date, many substances have been assessed for the delivery of nucleic acids, including CPPs, but there is still a lack of efficient, safe, and specific methods for the *in vivo* delivery of potentially therapeutic nucleic acids, including miRNA mimics.^71^, ^72^ We previously demonstrated the capacity of CPP‐miR‐146a noncovalent nanocomplexes to suppress inflammatory responses in cell cultures and a mouse model of irritant contact dermatitis.^73^ To our knowledge, we report for the first time that intranasal administration of CPP‐miRNA nanocomplexes is efficient for delivering miRNA mimics into mouse airways.

One of the main concerns in the development of nucleic acid‐based therapies is off‐target effects. However, as miRNA‐s are designed by nature, it has been suggested that there are fewer off‐target effects caused by the binding of either strand of miRNA mimics to unexpected targets than artificially designed siRNAs.^74^ In our study, we also assessed the presence of the Cy5 signal in frozen sections of organs collected at the end of the experiment; however, we did not detect any signal in the kidneys, spleen, liver, or MLN sections (data not shown). This suggests that most of the CPP‐miRNA nanocomplexes accumulated in the lungs, and therefore, there is less chance for off‐target effects.

It should be noted that several open questions concerning the functions of miR‐146a/b in the regulation of cellular responses of HBECs and immune responses during airway inflammation remain. For example, we previously reported reduced miR‐146a expression in bronchial brushings from asthma patients. Therefore, it would be interesting to study miR‐146 family expression and function during RV infection in HBECs from asthma patients. Another limitation of our study is that all experiments in HBECs were performed in submerged undifferentiated HBECs, and this did not enable us to assess the influence of miR‐146a/b on the differentiation of HBECs. Yet another question is whether miR‐146a/b affects all types of RVs. For example, several recent studies have revealed the importance of RV‐C in the development and exacerbation of asthma in children.^75^, ^76^, ^77^ Our study did not include RV‐C; however, similar to the included RV species, increased secretion of CXCL10 and IFN‐λ in response to RV‐C has been reported from human primary nasal epithelial cells.^78^ This indicates that the cellular responses of epithelial cells are similar in the case of RV‐A16, RV‐A1b, and RV‐C and that miR‐146a/b may also act as an anti‐inflammatory miRNA during RV‐C infection. Similar to the cell culture experiments, many questions remain to be studied regarding the influence of miR‐146a/b on immune responses in mouse models. For example, although we demonstrated increased infiltration of Th1/Th17 cells in the BAL of *Mir146a/b^−/−^* mice subjected to RV‐induced exacerbation of allergic airway inflammation, further studies are needed to better describe the individual effects of miR‐146a/b in different cell types. Accordingly, to confirm changes in Th1/Th17/Th2 balance, it would be interesting to repeat the experiment with antibodies for transcription factors GATA‐3, RORgt, and T‐bet to distinguish effector T cell subsets in flow cytometry.^79^, ^80^, ^81^ Similarly, it would be also interesting to assess the effect of miR‐146a/b deficiency in a case when RV infection is performed simultaneously with antigen challenge. In addition, it would be interesting to investigate the suppressor capacity of Treg cells in *Mir146a/b^−/−^* mice, as previously, Treg cells from *Mir146a^−/−^* mice were shown to be defective in their capacity to suppress Th1 responses.^64^ Also, since *Mir146a/b^−/−^* mice displayed impaired Th2 responses in the mouse models of HDM‐induced airway inflammation and RV‐induced exacerbation of allergic airway inflammation, it would be interesting to study whether there is an effect on type 2 ILC2, which likewise to Th2 cells, are capable of producing type 2 cytokines, including IL‐4, IL‐5, and IL‐13^82^, ^83^ and may therefore compensate for the reduced effect of Th2 cells.

Finally, as CPP‐miR‐146a nanocomplexes had an effect when applied after, but not before the challenge, our data suggest that these complexes penetrate to the tissue only during active inflammation and are therefore suitable for treatment, but not for prevention. Also, in the current study, the therapeutic potential of intranasal administration of CPP‐miRNA nanocomplexes was analyzed in the HDM‐induced airway inflammation model; however, the effect in the mouse model of RV‐induced exacerbation of allergic airway inflammation remains to be explored. Furthermore, it would be interesting to study whether continued treatment with CPP‐miRNA nanocomplexes would have a stronger anti‐inflammatory effect in the airways when compared with administration of nanocomplexes during the HDM challenge phase only. Therefore, although these first results are promising, additional preclinical experiments are clearly needed to assess the safety and optimize the methods of applying CPP‐miRNA nanocomplexes to target airway inflammation before any clinical trials could be initiated.

In conclusion, in the current study, we demonstrated that miR‐146a/b exerts anti‐inflammatory properties in HBECs and mouse airways during RV infection. Furthermore, even though the lack of miR‐146a/b led to reduced inflammation in murine HDM‐induced airway inflammation and RV‐induced exacerbation models, the intranasal application of CPP‐miR‐146a nanocomplexes strongly inhibited allergic inflammation in mouse airways, suggesting that the temporary overexpression of miR‐146a may exert a therapeutic effect in targeting different airway inflammation conditions.

## 4 MATERIALS AND METHODS

### 4.1 Isolation and culture of HBECs

HBECs were isolated by initial short‐term pronase (Roche, Basel, Switzerland) and DNase (Sigma‐Aldrich) digestion of bronchoscopy biopsies obtained from nonasthmatic donors from the Department of Pulmonology, Jagiellonian University Medical College (Krakow, Poland). In total, HBECs from two nonasthmatic donors were obtained (one male and one female Caucasian of age 26 and 30, respectively). Both donors underwent diagnostic bronchoscopy, and chronic airway disease was ruled out during further analyses. Frozen HBECs of passage 0 were thawed and cultured in BEGM Bulletkit (Lonza, Switzerland) media containing bovine pituitary extract, insulin, hydrocortisone, gentamicin and amphotericin‐B, retinoic acid, transferrin, triiodothyronine, epinephrine, and human epidermal growth factor as recommended by the manufacturer, and cells were transferred to a cell culture incubator (at 37°C under 5% CO_2_) for further experiments. This study was approved by the Ethics Committee of the Jagiellonian University Medical College.

### 4.2 Culture and maintenance of RV stocks

RV‐A16‐GFP was generated from the pA16‐eGFP plasmid by reverse genetics as previously described.^84^, ^85^ To propagate RV‐A16‐GFP and other RV serotypes used in the study, A16 and A1b RVs were cultured in Ohio HeLa cells.^35^ All virus stocks from crude cell lysates were titrated by standard methods using HeLa monolayers in RPMI media and serially diluted RVs to estimate TCID_50_/ml by the Kremser method and stored at ‐80°C until further use.

### 4.3 Infection and transfection of HBECs

For experiments in HBECs, 3 × 10^4^ cells per well were seeded into 12‐well plates (Greiner Bio‐one, Germany). Twenty‐four hours later, BEGM media from plated cells was replaced with fresh media, and cells were infected with RV‐A16, RV‐A1b, or RV‐A16‐GFP at a multiplicity of infection (MOI) of 0.1 or a negative control "mock," containing RPMI media from HeLa cells without RV particles. An MOI of 0.1 was chosen as one of the lowest concentrations previously shown to infect and activate HBECs^86^ and because it was sufficient to induce cellular responses of HBECs (Figure S1). For transfection, HBECs were seeded into 12‐well plates at a density of 4 × 10^4^ cells per well, and 24 h later, 30 nM miRIDIAN microRNA Mimic Negative Control #1 (Dharmacon, USA) or miRIDIAN microRNA hsa‐miR‐146a‐5p mimic (Dharmacon, USA) was transfected using MIRFECT (RNAexact, Estonia) according to the manufacturer's protocol. Twenty‐four hours after transfection, HBECs were infected with the indicated RVs or negative control mock. The supernatant and cells were harvested 24 h or 48 h later for further assays. For harvesting RNA, 500 μl Qiazol (Qiagen, Germany) was added to cells and stored at ‐20°C until RNA isolation.

### 4.4 RNA extraction, cDNA synthesis, and RT‐qPCR

To isolate RNA from HBECs and mouse lung lobes, a Total RNA Zol‐Out kit (A&A Biotechnology, Poola) and a Total RNA Zol‐Out D kit (A&A Biotechnology, Poola) were used according to the manufacturer's guidelines, respectively. To analyze relative miRNA and mRNA expression in cells and mouse lungs, RT‐qPCR and ΔΔCt calculations were used. As housekeeping genes for normalization, let7a was used for miRNA analysis, and EEF1A1 or Hprt was used for mRNA analysis. The data were compared relative to the mean value of the control group or condition, which was normalized to 1 and is indicated in each figure legend. More detailed descriptions of RNA isolation, cDNA synthesis, RT‐qPCR, and primer sequences are included in this article's Supplementary Information.

### 4.5 Neutrophil chemotaxis assay

Primary human neutrophils were isolated from a healthy donor's whole blood essentially as previously described^87^ using gradient centrifugation on Ficoll‐Paque Plus (GE Healthcare, Chicago, IL, United States). To lyse erythrocytes, red blood cell lysis buffer (Merck, Darmstadt, Germany) was used. Neutrophils were seeded at a density of 4 × 10^5^ on ThinCert cell culture inserts (3‐μm pore size) (Greiner Bio‐One, Kremsmünster, Austria) and placed into 24‐well plates. The outer chamber contained supernatants from HBECs transfected with miRNA mimics and stimulated with cytokines. Sixty minutes after incubation at 37°C under 5% CO2, the number of neutrophils migrating from the insert into the outer chamber supernatant was analyzed using a BD LSRFortessa (BD Biosciences, USA) cell analyzer. For each sample, the number of events was recorded for 1 min.

### 4.6 Mouse lines

All mice were bred and maintained under specific pathogen‐free conditions in the Laboratory Animal Center of the University of Tartu (Tartu, Estonia). *The Mir146a/b^−/−^* mouse line was created by crossing *Mir146b^−/−^* and *Mir146a^−/−^* lines in the Laboratory Animal Center of the University of Tartu (Tartu, Estonia). *Mir146b^−/−^* mice were generated as previously described.^24^ C57Bl/6J *wild‐type* (*wt*) and *Mir146a^−/−^* mice were purchased from Jackson Laboratory (Bar Harbor, US). Six‐ to ten‐week‐old mice were used in all experiments. No significant behavioral or phenotypical differences between *Mir146a/b‐/‐* and *wt* mice were observed during maintenance.

### 4.7 Mouse disease models

All animal studies were approved by the Animal Ethics Committee at the Ministry of Agriculture Estonian Government (01.03.2018, license 117). A mouse model of RV‐A1b‐induced airway inflammation was established as previously described.^35^ Briefly, C57Bl/6J *wt* and *Mir146a/b^−/‐^* mice were lightly anesthetized with isofluorane, and 40 μl RV‐A1b (7.78 × 10^5^ TCID_50_) or PBS as a control was administered i.n. Twenty‐four hours after infection, mice were sacrificed, and BAL fluid and the left upper lobe of the lung were collected from each mouse.

Mouse models of HDM‐induced allergic airway inflammation and RV‐A1b‐induced exacerbation of allergic airway inflammation were developed based on previous studies.^35^, ^36^, ^37^ In both models, 1 μg HDM extract (Greer Laboratories, Lenoir, NC) in 40 μl PBS was administered i.n. on day 0 to sensitize the C57Bl/6J *wt* and *Mir146a/b^−/−^* mice. On day 7–11, mice were challenged daily with 10 μg HDM extract in 40 μl of PBS (controls received PBS only) to induce allergic airway inflammation. On day 21 mice were i.n. infected with 40 μl RV‐A1b (7.78 × 10^5^ TCID_50_) or PBS as a control. Mice were sacrificed on day 22, and BAL fluid and lung tissue were collected for further analysis.

The effect of CPP‐miR‐146a nanocomplexes was assessed in a mouse model of HDM‐induced allergic airway inflammation.^36^, ^37^ For that, C57Bl/6J *wt* mice were lightly anesthetized, and for sensitization, 1 μg HDM extract in 40 μl PBS was administered i.n. to mice on day 0. On day 7–11, 10 μg HDM extract in 40 μl PBS was administered daily, and 2 h later, 60 pmol CPP‐miR‐146a or CPP‐control miR mimic nanocomplexes in 40 μl PBS were administered i.n. On day 15, mice were sacrificed, and blood, BAL fluid, and lung lobes were collected. More detailed information can be found in the Supplementary Materials and Methods.

### 4.8 Collection and analyses of BAL fluid

BAL fluid samples were obtained by lavaging the airways of mice three times with 1 ml of PBS through a tracheal cannula. First, 1 ml of collected BAL was collected into separate tubes and centrifuged at 300 rcf for 5 min, and supernatants were used for protein quantification using ELISA. Next, erythrocytes were lysed using Red Blood Cell Lysis Buffer (Roche, Basel, Switzerland), and BAL cells were counted and stained with fluorochrome‐labeled antibodies according to the manufacturer's instructions and subjected to flow cytometry analysis using BD LSRFortessa (BD Biosciences, USA). For each sample, 50,000 events were recorded, and data were analyzed using the FCS Express 7 program. The gating strategy used to identify BAL cells is shown in Figure S5. A more precise sorting protocol appears in the Supplementary Materials and Methods section.

### 4.9 Isolation and stimulation of mouse splenocytes and BMDCs

Single splenocyte suspensions were prepared from *wt* and *Mir146a/b^−/−^* mouse spleens by homogenization through 100 μm cell strainers followed by washing in RPMI‐1640 medium (Merck) supplemented with 10% FBS and 1% Pen‐Strep. To obtain DCs, bone marrow cells from *wt* and *Mir146a/b^−/−^* mice were isolated and cultured in RPMI‐1640 medium supplemented with 1% FBS and 20 ng/ml GM‐CSF as previously described.^88^ For stimulation, 50 μg HDM, 100 μl of RV‐A1b (TCID_50_ = 1.53*10^4^) or 50 μg HDM + 100 μl of RV‐A1b (TCID_50_ = 1.53*10^4^) was added to 2  ×  10^6^ splenocytes in 500 μl of RPMI for 48 h or to 5  ×  10^5^ BMDCs per well for 24 h. As a negative control, splenocytes were left unstimulated or 100 μl of "mock" medium containing RPMI medium from uninfected HeLa cells was used.

### 4.10 Protein quantification

Protein levels in the supernatants of HBECs or BAL fluid were analyzed by ELISA. For HBEC samples, human IL‐8 ELISA MAX Deluxe Set (BioLegend, 431504), human CXCL1/GRO alpha DuoSet ELISA (R&D Systems, DY275‐05), and human IL‐29/IL‐28B (IFN‐lambda 1/3) DuoSet ELISA kit (R&D Systems, DY1598B‐05) were used according to the manufacturers’ instructions. For BAL fluids, a mouse CXCL1/KC DuoSet ELISA kit (R&D Systems, DY453‐05) was used as instructed by the manufacturer. The data were acquired with a Ledetect 96 Microplate reader (Labexim Products, Lengau, Austria). Online analysis software Myassays.com and a four‐parameter logistic regression model were used to calculate the concentrations of the proteins.

### 4.11 Target and pathway analysis

One thousand eighty one unconserved targets with cumulative weighted context score < −0.1 were selected with TargetScan 7.2^32^ and compared with the dataset of 4647 genes detected to be differentially expressed in response to human RVs in HBECs from non‐asthmatic individuals.^33^ Three hundred fifty overlapped genes we subjected to pathway analysis using enrichr^89^ and the MSigDB Hallmark Gene Set Collection.^34^

### 4.12 Statistical analysis

GraphPad Prism 6 (GraphPad Software Inc., USA) and unpaired two‐sided Student's *t*‐test or one‐way ANOVA with Tukey's multiple comparisons test were used for visualization and statistical analysis, as indicated in the figure legends. The results were considered to be significant at *p < 0.05; **p < 0.01; ***p < 0.001.

## CONFLICT OF INTEREST

Ana Rebane and Margus Pooga are board members of RNAexact OÜ. All other authors declare no conflict of interest regarding this manuscript.

## AUTHOR CONTRIBUTIONS

Anet Laanesoo performed the experiments, analyzed the data, contributed to the design of the study, and wrote the manuscript. Egon Urgard and Jonathan Jonathan M. Coquet contributed to the design and performance of experiments in mice. Kapilraj Periyasamy performed experiments in mouse splenocytes and BMDCs. Yury A. Bochkov, James E. Gern, Alar Aab, and Sebastian L. Johnston provided GFP‐tagged and wt RV strains and contributed to the study design. Jesper Wengel designed and provided miRNA mimics for mouse experiments. Grazyna Bochenek and Bogdan Jakiela prepared HBECs and contributed to the study design. Mark P. Boldin and Nathaniel Magilnick provided knockout mouse lines. Martti Laan, Margus Pooga, and Alan Altraja contributed to the study design and review of the manuscript. Ana Rebane designed the study, supervised the project, and wrote the manuscript. All authors read and approved the final version of the manuscript.

## Supporting information

Supporting information

## Data Availability

The datasets used and analyzed during the current study are available from the corresponding author on reasonable request.
