# Supplementary material for: Dual role of the miR‐146 family in rhinovirus‐induced airway inflammation and allergic asthma exacerbation
Source: Clin Transl Med. 2021 May 28;11(6):e427. doi: 10.1002/ctm2.427 (PMC8161513; doi:10.1002/ctm2.427)
Supplement: Supplementary file 1 — Supporting information [file CTM2-11-e427-s001.pdf]

Supplementary

# Dual role of the miR-146 family in rhinovirus-induced airway inflammation and allergic asthma exacerbation

Anet Laanesoo, MSc[1], Egon Urgard, PhD[1], Kapilraj Periyasamy, MSc[1], Martti Laan, MD, PhD[1], Yury A. Bochkov, PhD[2], Alar Aab, PhD[1], Nathaniel Magilnick, PhD[3], Margus Pooga, PhD[4], James E. Gern, MD[2], Sebastian L. Johnston, MD, PhD[5,6], Jonathan M. Coquet, PhD[7], Mark P. Boldin, MD, PhD[3], Jesper Wengel PhD[8], Alan Altraja, MD, PhD[9,10], Grazyna Bochenek, MD, PhD[11], Bogdan Jakiela, MD, PhD[11] and Ana Rebane, PhD[1]

[1] Institute of Biomedicine and Translational Medicine, University of Tartu, Estonia

[2] School of Medicine and Public Health University of Wisconsin-Madison, Madison, Wisconsin

[3] Department of Molecular and Cellular Biology, Beckman Research Institute of City of Hope National Medical Center, Duarte, California, USA

[4] Institute of Technology, University of Tartu, Estonia

[5] National Heart and Lung Institute, Imperial College London, London, United Kingdom

[6] Imperial College Healthcare NHS Trust, London, United Kingdom

[7] Department of Microbiology, Tumor and Cell Biology (MTC), Karolinska Institutet, Stockholm, Sweden

[8] Nucleic Acid Center, Department of Physics, Chemistry and Pharmacy, University of Southern Denmark, Denmark

[9] Department of Pulmonary Medicine, University of Tartu, Estonia

[10] Lung Clinic of the Tartu University Hospital, Estonia

[11] Department of Medicine, Jagiellonian University Medical College, Krakow, Poland

Corresponding author: Ana Rebane, Institute of Biomedicine and Translational Medicine, University of Tartu, Ravila 14B, 50414 Tartu, Estonia; E-mail: ana.rebane@ut.ee

**Supplementary Table S1.** miR-146a putative target genes modulated by RVs are associated with immune regulatory pathways*

| Term | Overlap | P-value | Adjusted P-value | Odds Ratio | Genes |
|---|---|---|---|---|---|
| Interferon Gamma Response | 14/200 | 0,00001 | 0,0006 | 4,36 | CD86; SAMD9L; STAT1; DDX58; UBE2L6; IFI35; BPGM; PTGS2; IFIT3; CASP7; PNP; APOL6; EPSTI1; ST8SIA4 |
| TNF-alpha Signaling via NF-kB | 11/200 | 0,00083 | 0,0191 | 3,34 | RCAN1; BTG2; SERPINB2; LAMB3; DDX58; ACKR3; MAP3K8; SLC2A3; PTGS2; MXD1; ETS2 |
| Interferon Alpha Response | 7/97 | 0,00158 | 0,0242 | 4,44 | SAMD9L; TRIM5; EPSTI1; HLA-C; UBE2L6; IFI35; IFIT3 |
| IL-2/STAT5 Signaling | 10/199 | 0,00274 | 0,0261 | 3,03 | CD86; SOCS2; NT5E; PNP; PLIN2; MAP3K8; SLC2A3; MXD1; HK2; CKAP4 |
| Complement | 10/200 | 0,00284 | 0,0261 | 3,01 | CTSO; CASP7; SERPINB2; KCNIP3; PREP; GNAI3; PRCP; RASGRP1; GNAI2; HSPA1A |

*Unconserved miR-146a targets with cumulative weighted context score < -0.1. were selected with TargetScan 7.2. [1] and compared with the dataset of differentially expressed genes in HBECs stimulated with RVs.[2] 350 overlapped genes we subjected to pathway analysis using enrichr [3] and the Molecular Signatures Database (MSigDB) Hallmark Gene Set Collection. [4]

## Construction of the pA16-GFP infectious clone

The full-length cDNA clone pR16.11, which produces infectious recombinant RV-A16 virus, was published [5]. We amplified the enhanced green fluorescent protein (eGFP) from the pcDNA3-GFP (Addgene), and partial 5’ untranslated region (5’UTR) and VP4/VP2 sequences of RV-A16 from pR16.11 cDNA template by PCR using the primers listed in Supplementary Table S2. Three PCR products were then purified by QIAquick PCR Purification Kit (Qiagen), combined and reamplified using the flanking primers RV1a16-f and NdeI-RV16-r. The resulting amplicon (1747 base pair long) was digested by BstAPI and NdeI and ligated with similarly digested pR16.11 to generate pA16-GFP clone. The eGFP sequence was inserted in frame between 5’ UTR and VP4 and followed by additional 2A protease recognition site (NLTTV / G) to release the GFP from viral polyprotein upon translation.

**Supplementary Table S2**. Primers used for construction of the GFP-expressing pA16-GFP infectious clone.

| Primer | Sequence (5'-3') | PCR product |
|---|---|---|
| RV1a16-fw<br>ATG-GFP16-rv | CCTCCGGCCCCTGAAT<br>TCCTCGCCCTTGCTCACAGCGCCCATGATAACAATATATATATG | 1 |
| ATG-GFP16-fw<br>2A-GFP16-rv | ATATTGTTATCATGGGCGCTGTGAGCAAGGGCGAGGAGCTGTTC<br>GCCAACAGTTGTTAGATTCTTGTACAGCTCGTCCATGCCGA | 2 |
| 2A-GFP16-fw<br>NdeI-RV16-rv | TACAAGAATCTAACAACTGTTGGCGCTCAAGTATCTAGACAG<br>GAACAGCATTAACATATGGTAC | 3 |

fw – forward primer; rv – reverse primer

**RNA extraction, cDNA synthesis and RT-qPCR**

The concentration and purity of isolated RNAs were assessed with NanoDrop 2000c (Thermo Scientific, USA) spectrophotometer.

To analyze miR-146a expression in transfected cells, TaqMan® MicroRNA Assays (Life Technologies, California, USA) and 5× HOT FIREPol® Probe qPCR Mix Plus (ROX) (Solis BioDyne, Tartu, Estonia) were used according to manufacturers' instructions. miRNA expression in RV stimulated HBECs and mouse lungs was determined by using miScript II RT Kit, miScript SYBR Green PCR Kit and Hs_miR-146a_1 miScript Primer Assay (cat. MS00003535) by Qiagen according to the manufacturer's protocol. Each PCR reaction was performed in duplicates using a ViiA 7 Real-Time PCR system (Life Technologies, Carlsbad, California, USA). To normalize miRNA expression, let-7a and ΔΔCt calculation were used. To determine mRNA expression, cDNA was synthesized using 200-500 ng of total RNA, oligo-dT (TAG Copenhagen, Denmark), RevertAid Reverse Transcriptase (Thermo Scientific) and RiboLock RNase Inhibitor (Thermo Scientific). For quantitative polymerase chain reaction, 5x HOT FIREPol EvaGreen qPCR Supermix (Solis BioDyne, Estonia) and ViiA™ 7 machine (Applied Biosystems) were used. The expression of target genes was normalized to EEF1A1 or Hprt expression using ΔΔCt calculation. The sequences of qPCR primers are provided in Supplementary Table S3.

**Supplementary Table S3.** Sequences of qPCR primers.

| | |
|---|---|
| hEEF1A1 | fw 5’ CCACCTTTGGGTCGCTTTGCTGT 3’<br>rev 5’ TGCCAGCTCCAGCAGCCTTCTT 3’ |
| hIRAK1 | fw 5’ CACCTTCAGCTTTGGGGTGGTAGTG 3’<br>rev 5’ CCAGCCTCCTCAGCCTCCTCT 3’ |
| hCARD10 | fw 5’ AGTGTGCCCAGCGGAAAGCC 3’<br>rev 5’ GATGGCCCGGATCCTGCTGC 3’ |
| hCCL5 | fw 5’ AGTCGTCTTTGTCACCCGAAA 3’<br>rev 5’ TCTCCATCCTAGCTCATCTCCAA 3’ |
| hIL-8 | fw 5’ GCAGCTCTGTGTGAAGGTGCAGTT 3’<br>rev 5’ TTCTGTGTTGGCGCAGTGTGGTC 3’ |
| hCXCL1 | fw 5’ TTGCCTCAATCCTGCATCCC 3’<br>rev 5’ GGTCAGTTGGATTTGTCACTGT 3’ |
| hIFITM1 | fw 5’ CAACACCCTCTTCTTGAACTGG 3’<br>rev 5’ GCCGAATACCAGTAACAGGATG 3’ |
| hIRF1 | fw 5’ CAACTTCCAGGTGTCACCCA 3’<br>rev 5’ CGACTGCTCCAAGAGCTTCA 3’ |
| hICAM-1 | fw 5’ TCAGTCAGTGTGACCGCAGA 3’<br>rev 5’ GCGCCGGAAAGCTGTAGATG 3’ |
| mHprt | fw 5’ CTCTCGAAGTGTTGGATACAG 3’<br>rev 5’ ACAAACGTGATTCAAATCCCC 3’ |
| mIrak1 | fw 5’ CCCCCATGACCCAGAGGCAAAAC 3’<br>rev 5’ TCCAGCAAAGCAGCAGCCCTT 3’ |
| mCard10 | fw 5’ GCGAGGTCTACCCCATTGTC 3’<br>rev 5’ CAACAGGCCCCTGATCTCAC 3’ |
| mCxcl1 | fw 5’ ACCGAAGTCATAGCCACACTC 3’<br>rev 5’ CTCCGTTACTTGGGGACACC 3’ |
| mCxcl2 | fw 5’ TGAACAAAGGCAAGGCTAACTG 3’<br>rev 5’ CAGGTACGATCCAGGCTTCC 3’ |

| | |
|---|---|
| mIrf1 | fw 5’ ACCAAGAGGAAGCTGTGTGG 3’<br>rev 5’ GCTGTGGTCATCAGGTAGGG 5’ |
| mIfitm1 | fw 5’ CCGTGAAGTCTAGGGACAGG 3’<br>rev 5’ GGAGCTGATGTTCAGGCACT3’ |
| mIfn-γ | fw 5’ TTCTTCAGCAACAGCAAGGC 3’<br>rev 5’ TGTGGGTTGTTGACCTCAAACT 3’ |
| mIl-4 | fw 5’ ATGGATGTGCCAAACGTCCT 3’<br>rev 5’ AAGCACCTTGGAAGCCCTAC 5’ |
| mIl-13 | fw 5’ GACCAGACTCCCCTGTGCAACG 3’<br>rev 5’ AGGGCTACACAGAACCCGCCA 5’ |
| mIl-17a | fw 5′ GTGTCAATGCGGAGGGAAA 3’<br>rev 5’ TTCAGGACCAGGATCTCTTGCT 3’ |
| mIl-33 | fw 5’ CCTGCAAGTCAATCAGGCGA 3’<br>rev 5’ GAGTAGTCCTTGTCGTTGGCA 3’ |
| mCcl11 | fw 5’ TGTCTCCCTCCACCATGCAGAGC 3’<br>rev 5’ TTGGGATGGAGCCTGGGTGAGC 3’ |
| mS100a8 | fw 5’ CTTCAAGACATCGTTTGAAAGG 3’<br>rev 5’ TCATTCTTGTAGAGGGCATGG 3’ |
| mS100a9 | fw 5’ CCATCAATACTCTAGGAAGGAAGG 3’<br>rev 5’ CTTCTCTTTCTTCATAAAGGTTGC 3’ |

h – human primer; m – mouse primer

**RV-A16-GFP**

To quantify RV-A16-GFP infected HBECs, HBECs were transfected with miRNA mimics and infected with RV-A16-GFP as described in section ’stimulation of HBEC with Rhinoviruses’. 48h after RV infection, the media was removed from HBECs, cells were washed once with PBS and removed from plate by using 2x Trypsin (Trypsin-EDTA in PBS, GE Healthcare, UK). Trypsinized HBECs were collected to new tubes, centrifuged at 4°C 250 rcf (Eppendorf 5424 R, rotor FA-45-24-11, Germany) for 5 min. Supernatants were removed, cells were resuspended in 1 ml PBS for washing and again centrifuged. Washing step was repeated 3 times. After last centrifugation HBECs were resuspended in 300 µl PBS and analyzed with BD LSRFortessa (BD Biosciences, USA). For each sample 10 000 events were recorded.

For fluorescence microcopy, RV-A16-GFP or mock was added to primary HBECs. 48h after stimulation cells were washed with PBS and stained with DAPI. The images were taken with Leica DM5500 B (Leica Microsystems, Wetzlar, Germany).

**Mouse models**

The effect of CPP-miR-146a nanocomplexes was assessed in a mouse model of HDM induced allergic airway inflammation [6,7]. For CPP-miRNA nanocomplexes 60 pmol of miRNA mimics (double stranded for miR-146a, Supplementary Table S4; or for unlabelled miRIDIAN Negative Control #1) at 28:1 CPP:miRNA molar ratio in 40 µl of PBS were administered. As delivering CPP, MIRFECT (RNAexact, Tartu, Estonia) was used. On day 15, mice were sacrificed and blood, BAL and lung lobes were collected. Similarly, to visualize the localization of CPP-miRNA nanocomplexes in mouse lung lobes, sulfo-cyanine5-maleimide (Lumiprobe #13380) coupled miR-146a mimics (Cy5-miR-146a) or CPP-control mimics were administered daily 2h after each HDM challenge. For CPP-miRNA nanocomplexes 60 pmol of miRNA mimics (double stranded for miR-146a-Cy5, Supplementary Table S4; or for unlabelled miRIDIAN Negative Control #1) at 28:1 CPP:miRNA molar ratio in 40 µl of PBS were administered i.n. As delivering CPP, MIRFECT was used. On day 11, lungs were fixed i.t. with 4% PFA for 15 min and collected lung lobe was further fixed for 24h at 4°C and then embedded into Tissue-Tek (Thermo Scientific, Waltham, USA) and frozen. Frozen lung lobes were cut to 10 µm sections, stained with DAPI and subjected to microscopy. Images from lung lobe sections were taken with confocal laser scanning microscope (Olympus FV1200MPE; Olympus Corporation, Japan) at 60x/1.40 oil objective at 1024x1024 pixel resolution.

**Supplementary Table S4.** Sequences of oligonucleotides used in *in vivo* administration*.

| | |
|---|---|
| ss miR-146a-5p | 5' rUrGrArGrArArCrUrGrArArUrUrCrCrArUrGrGrGrUrU 3' |
| ss Cy5-miR-146a-5p | 5' rUrGrArGrArArCrUrGrArArUrUrCrCrArUrGrGrGrUuU 3' + Cy5 |
| ss miR-146a-3p | 5' rCrCrCuArUrGrGrArArUrUrCrArGrGrUrCrArCrArUuU 3' |

* r – ribonucleotide, u – nucleotide contains unlocked ribose

**Collection of bronchoalveolar lavage**

BAL cells were then counted and stained with fluorochrome labelled antibodies according to manufacturer's instructions and subjected to flow cytometric analysis. CD4, ST2, CCR6 specific antibodies were purchased from Biolegend (San Diego, US), CD44, CXCR3, B220, CD3e, Ly6G and Siglec-F specific antibodies from BD Biosciences (New Jersey, US) and

FOXP3, CD11c antibodies from eBioscience (San Diego, US). Monocytes/macrophages were identified as $CD11c^{+}SiglecF^{+}$ cells, dendritic cells as $CD11c^{+}SiglecF^{-}$, neutrophils as $CD11c^{-}$ $Ly6G^{+}$, eosinophils as $CD11c^{-}Siglec\ F^{+}$, B-lymphocytes as $CD3^{-}B220^{+}$, regulatory T-cells as $CD4^{+}CD44^{-}FOXP3^{+}$, memory T-cells as $CD4^{+}CD44^{+}FOXP3^{-}$ and Th1 cells as $CD4^{+}CD44^{+}FOXP3^{-}CXCR3^{+}$, Th2 cells as $CD4^{+}CD44^{+}FOXP3^{-}ST2^{+}$, Th17 cells as $CD4^{+}CD44^{+}FOXP3^{-}CCR6^{+}$.

For differential staining BAL cells were processed by Shandon 2 cytospin device (Shandon Southern Instruments, Runcorn, UK) and slides were stained with Shandon™ Kwik-Diff™ Stains (ThermoFisher Scientific, Waltham, Massachusetts, US) and cell counts were performed, the absolute numbers and percentages of eosinophils, neutrophils, lymphocytes and macrophages were quantified.

**Supplementary references**


1 Agarwal, V., Bell, G. W., Nam, J. W. & Bartel, D. P. Predicting effective microRNA target sites in mammalian mRNAs. *Elife* **4**, doi:10.7554/eLife.05005 (2015).

2 Helling, B. A. *et al.* Altered transcriptional and chromatin responses to rhinovirus in bronchial epithelial cells from adults with asthma. *Commun Biol* **3**, 678, doi:10.1038/s42003-020-01411-4 (2020).

3 Kuleshov, M. V. *et al.* Enrichr: a comprehensive gene set enrichment analysis web server 2016 update. *Nucleic Acids Res* **44**, W90-97, doi:10.1093/nar/gkw377 (2016).

4 Liberzon, A. *et al.* The Molecular Signatures Database (MSigDB) hallmark gene set collection. *Cell Syst* **1**, 417-425, doi:10.1016/j.cels.2015.12.004 (2015).

5 Bochkov, Y. A. *et al.* Cadherin-related family member 3, a childhood asthma susceptibility gene product, mediates rhinovirus C binding and replication. *Proceedings of the National Academy of Sciences of the United States of America* **112**, 5485-5490, doi:10.1073/pnas.1421178112 (2015).

6 Tibbitt, C. A. *et al.* Single-Cell RNA Sequencing of the T Helper Cell Response to House Dust Mites Defines a Distinct Gene Expression Signature in Airway Th2 Cells. *Immunity* **51**, 169-184, doi:10.1016/j.immuni.2019.05.014 (2019).

7 Rodriguez-Perez, N. *et al.* Altered fatty acid metabolism and reduced stearoyl-coenzyme a desaturase activity in asthma. *Allergy: European Journal of Allergy and Clinical Immunology* **72**, 1744-1752, doi:10.1111/all.13180 (2017).

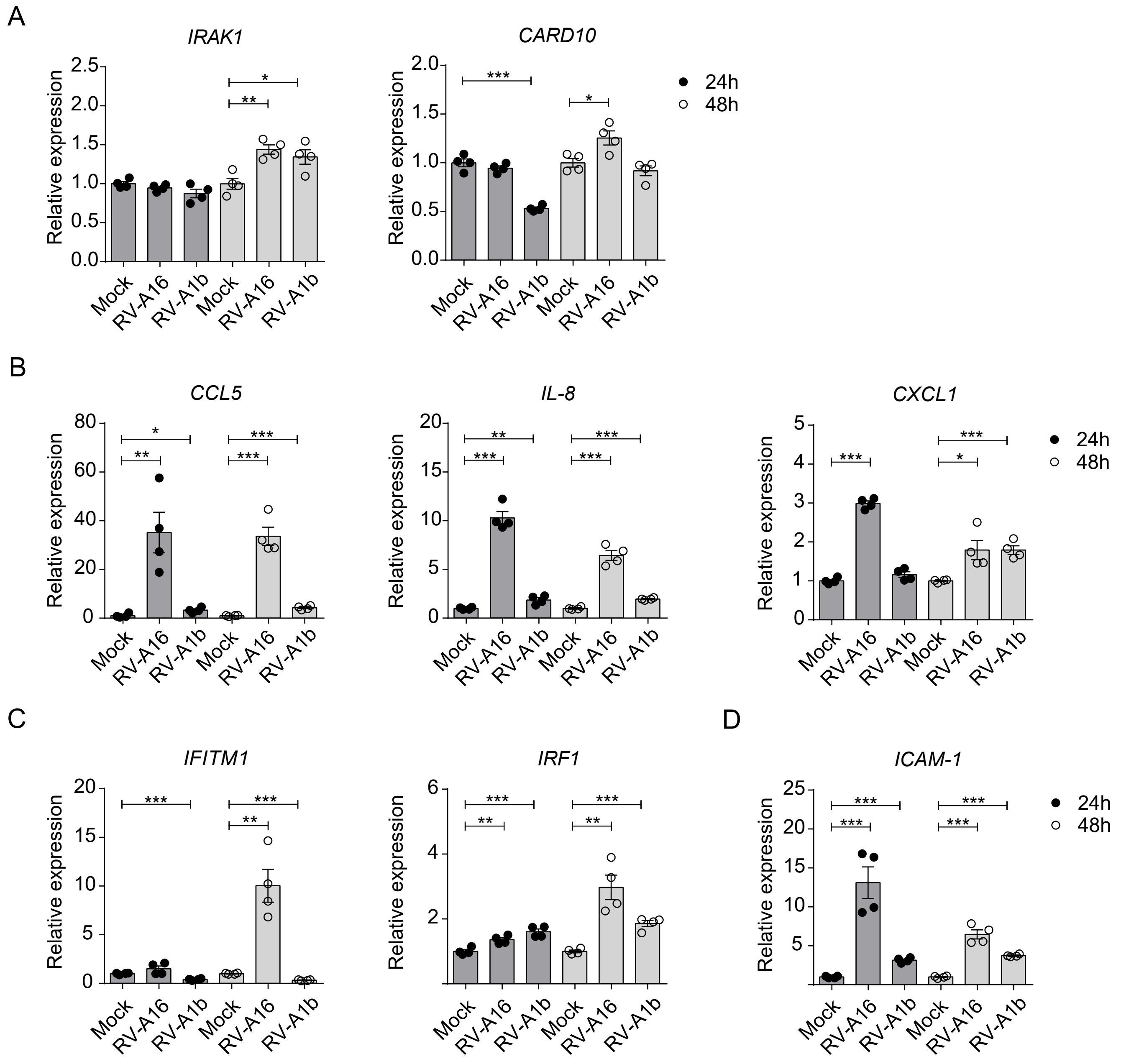


**Figure S1. RV infection stimulates the expression of NF-κB-inducible and interferon response genes in HBECs.** HBECs were stimulated with indicated RV strains for 24h or 48h. (A-D) mRNA expression of indicated miR-146a/b direct target genes (A), indirect target genes (B), interferon response genes (C), and RV-A16 receptor gene (D) in HBECs was measured by RT-qPCR and is shown in comparison with mock stimulated cells. Data represent mean ± SEM. Unpaired t-test, * $P < 0.05$, ** $P < 0.01$, *** $P < 0.001$.

A

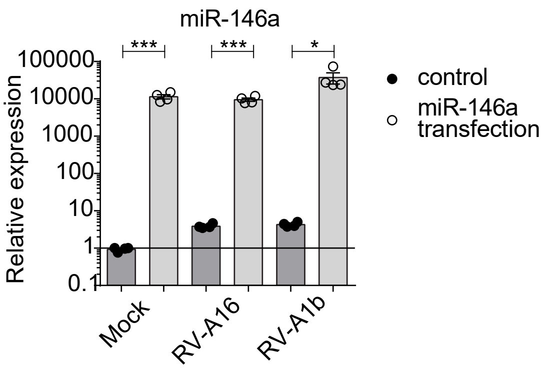


**Figure S2. Transfection of CPP-miR-146a complexes into HBECs results in overexpression of miR-146a.** HBECs were transfected with miRNA mimics for 24h and stimulated with indicated RVs or mock for 48h. miRNA expression was determined by RT-qPCR. Data represent mean ± SEM. Unpaired t-test, * $P < 0.05$, ** $P < 0.01$, *** $P < 0.001$.

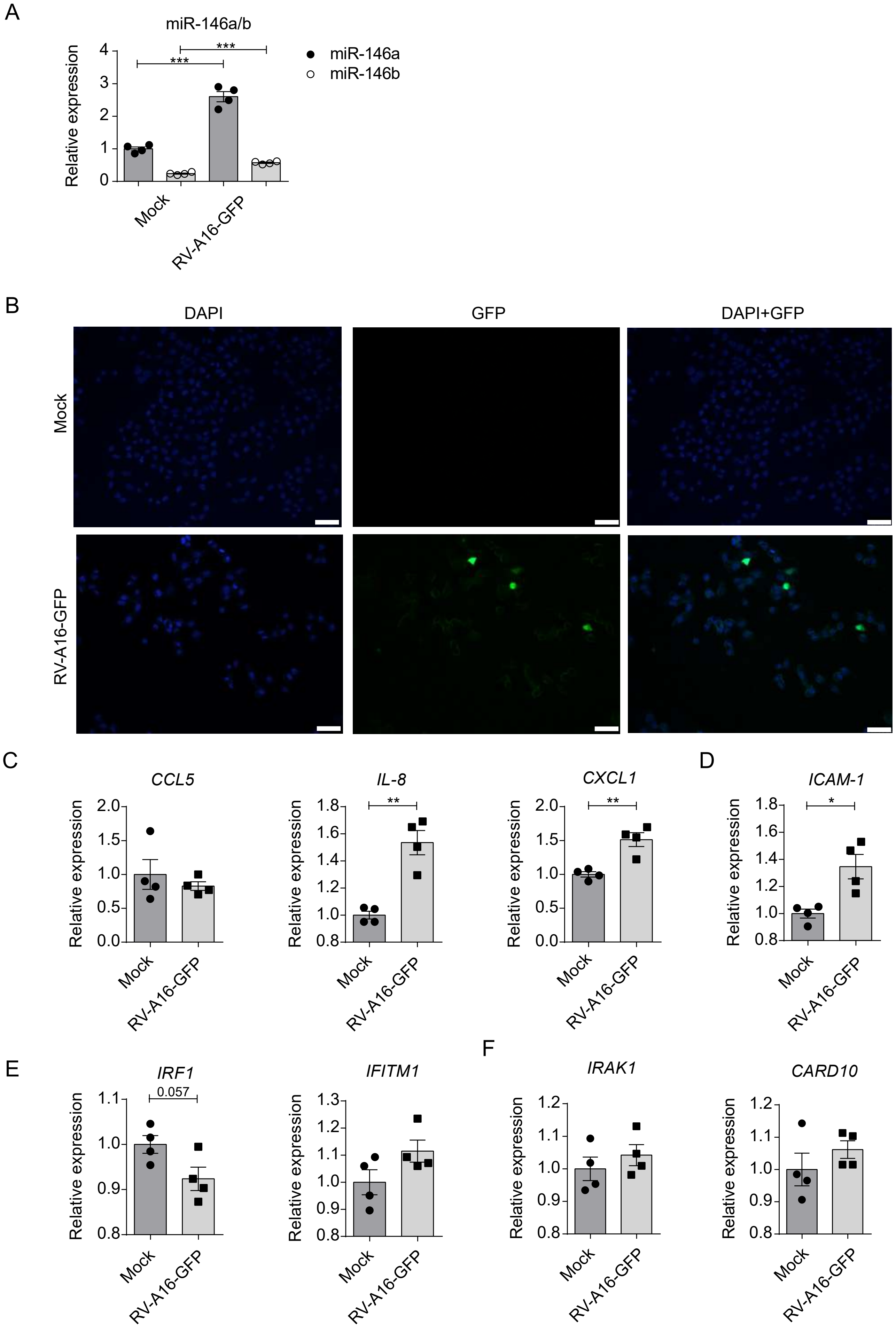


**Figure S3. RV-A16-GFP infection increases the expression of miR-146a/b and pro-inflammatory genes in HBECs**. Primary HBECs were stimulated with RV-A16-GFP for 48h or mock as control. Relative miRNA (A) and mRNA (C-F) expression in HBECs was measured by RT-qPCR and is shown in comparison with mock stimulated cells. Data represent mean ± SEM. Unpaired t-test, * $P < 0.05$, ** $P < 0.01$, *** $P < 0.001$. (B) 48h after RV stimulation, cells were counterstained with DAPI and subjected to fluorescence microscopy. Bar = 50 µm. One representative image of 3 replicates is shown.

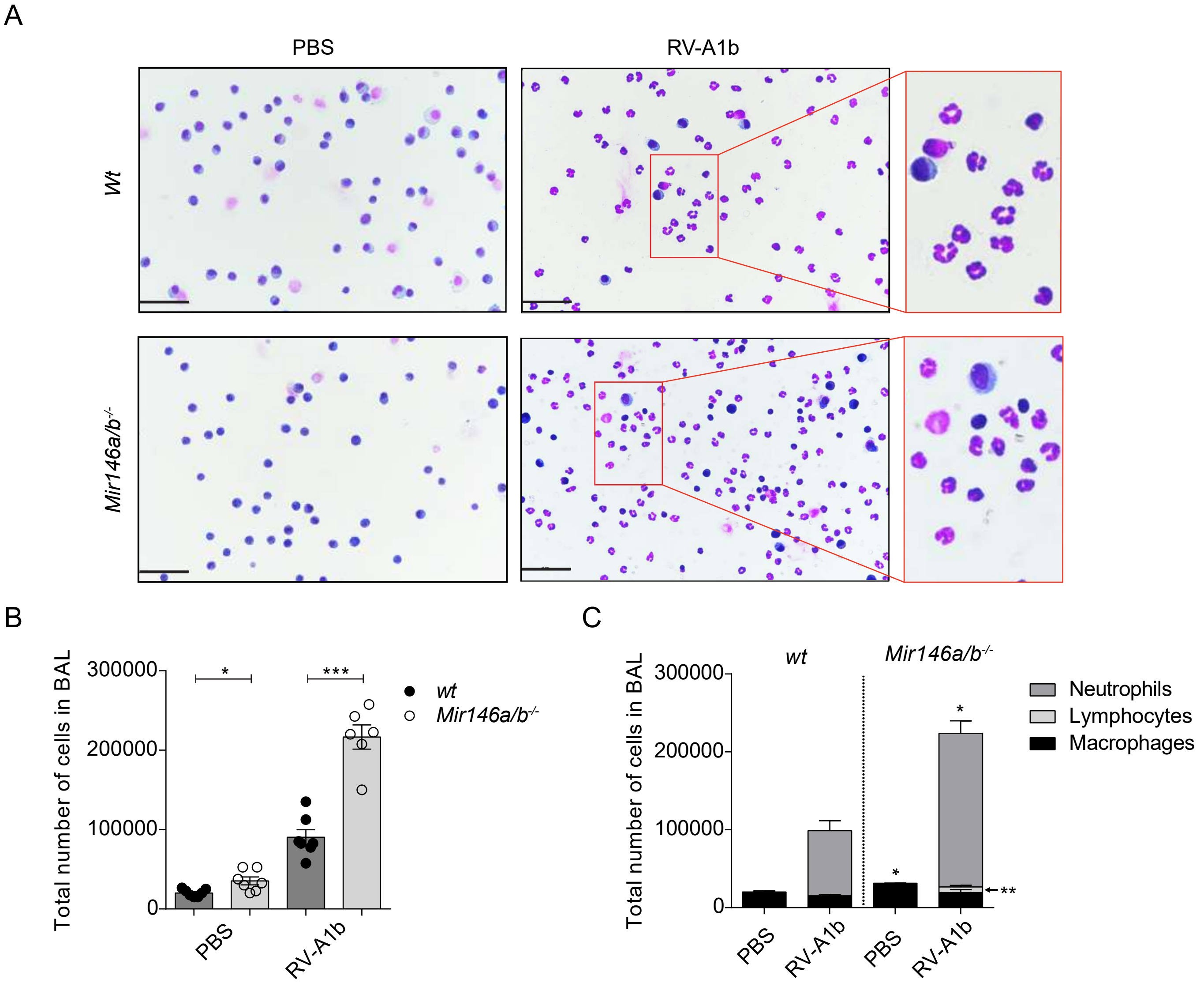


**Figure S4. Differential staining of BAL cells revealed an increased number of neutrophils in the airways of *Mir146a/b*$^{-/-}$ mice during RV infection.** RV-A1b or PBS was administered i.n. to *wt* or *Mir146a/b*$^{-/-}$ mice for 24h. Data from 3-4 mice in each group from 2 independent experiments are shown and represented as mean ± SEM. Unpaired t-test, * $P< 0.05$, ** $P < 0.01$, *** $P < 0.001$. BAL cells were counted using a hemocytometer (B), then cytospinned on glass slides and differentially stained (A). (C) Quantification of differentially stained BAL cells. Cells from PBS or RV-A1b treated *Mir146a/b*$^{-/-}$ mice were compared with the same *wt* treatment group.

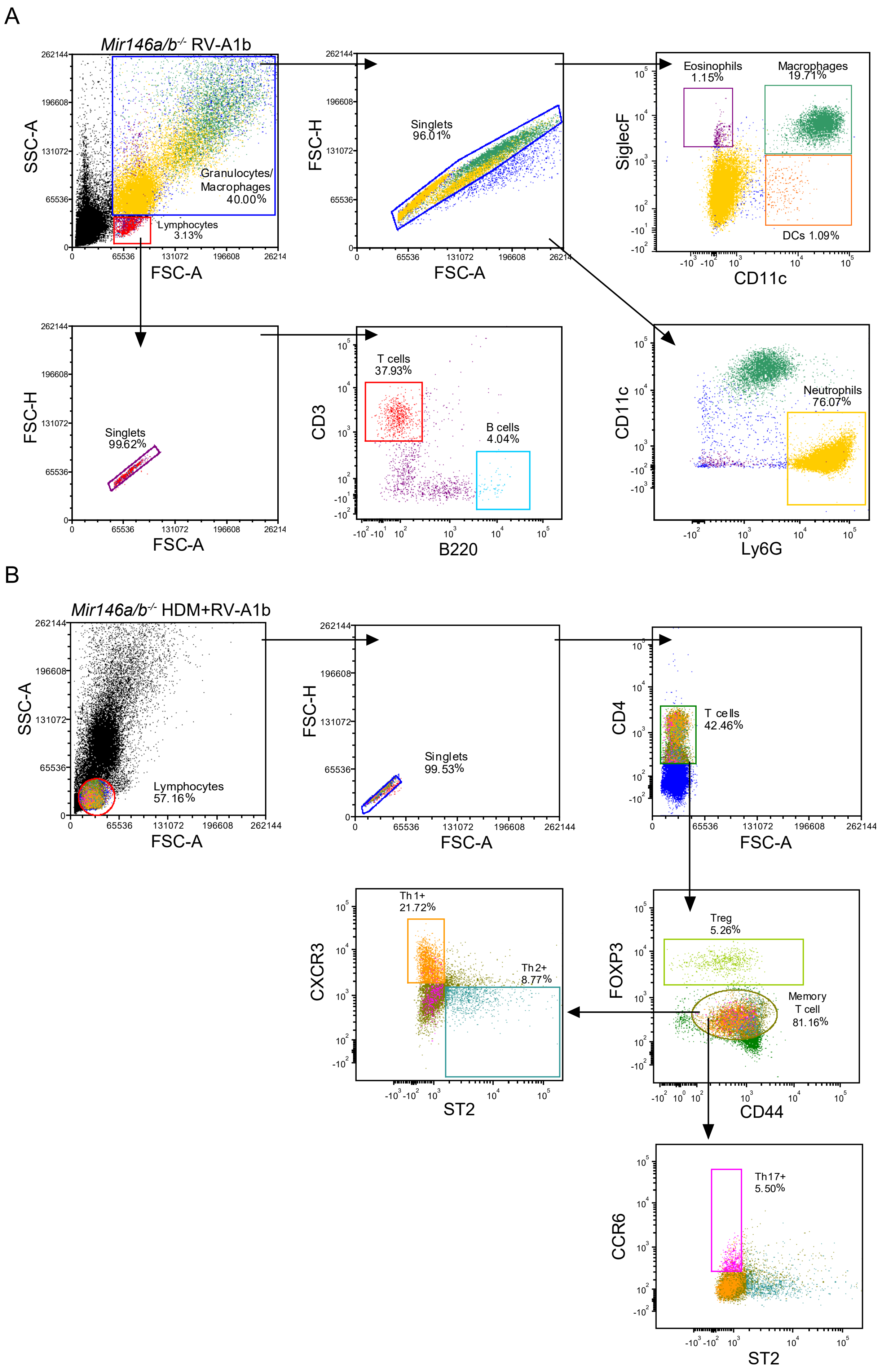


**Figure S5. Gating strategy for flow cytometry analysis of BAL cells.** (A) BAL cells were first gated by their size (SSC-A vs FSC-A) to separate granulocyte/macrophage and lymphocyte populations. Both populations were then gated for singlets (FSC-H vs. FSC-A) and further analyzed for their expression of Siglec F, CD11c, Ly6G or CD3, and B220. (B) Flow cytometry analysis of T helper cell subsets. Cells were first gated by their size (SSC-A vs FSC-A) to lymphocyte population, then gated for singlets (FSC-H vs FSC-A) and further analyzed for their expression of CD4, FOXP3 or CD44 and CXCR3, ST2, CCR6.

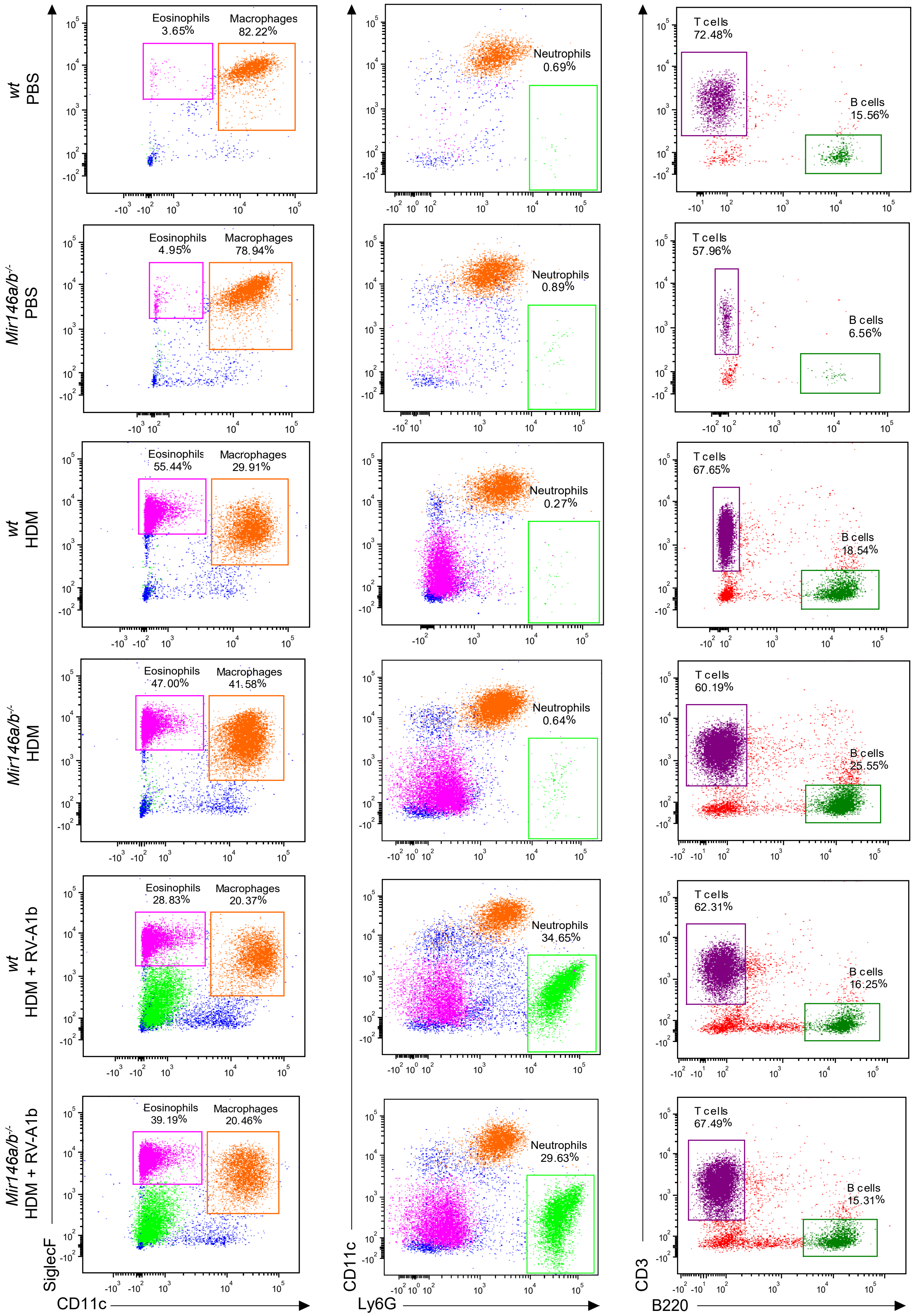


**Figure S6. Lack of miR-146a/b in mice results in an altered immune response in allergic airway inflammation and RV-induced asthma exacerbation models.** Mice were sensitized (D0) and challenged (D7-11) with HDM extract and when indicated, infected with RV-A1b (D21) for 24h. Control mice received only PBS. (A) One representative FACS dot-plot of BAL from three PBS treated, five HDM treated, and five HDM+RV-A1b treated *wt* and *Mir146a/b*$^{-/-}$ mice are shown. Eosinophils and neutrophils (left and middle panel) were analyzed as a percentage of granulocyte population and T cells, B cells were analyzed as a percentage of lymphocyte population (right panel).

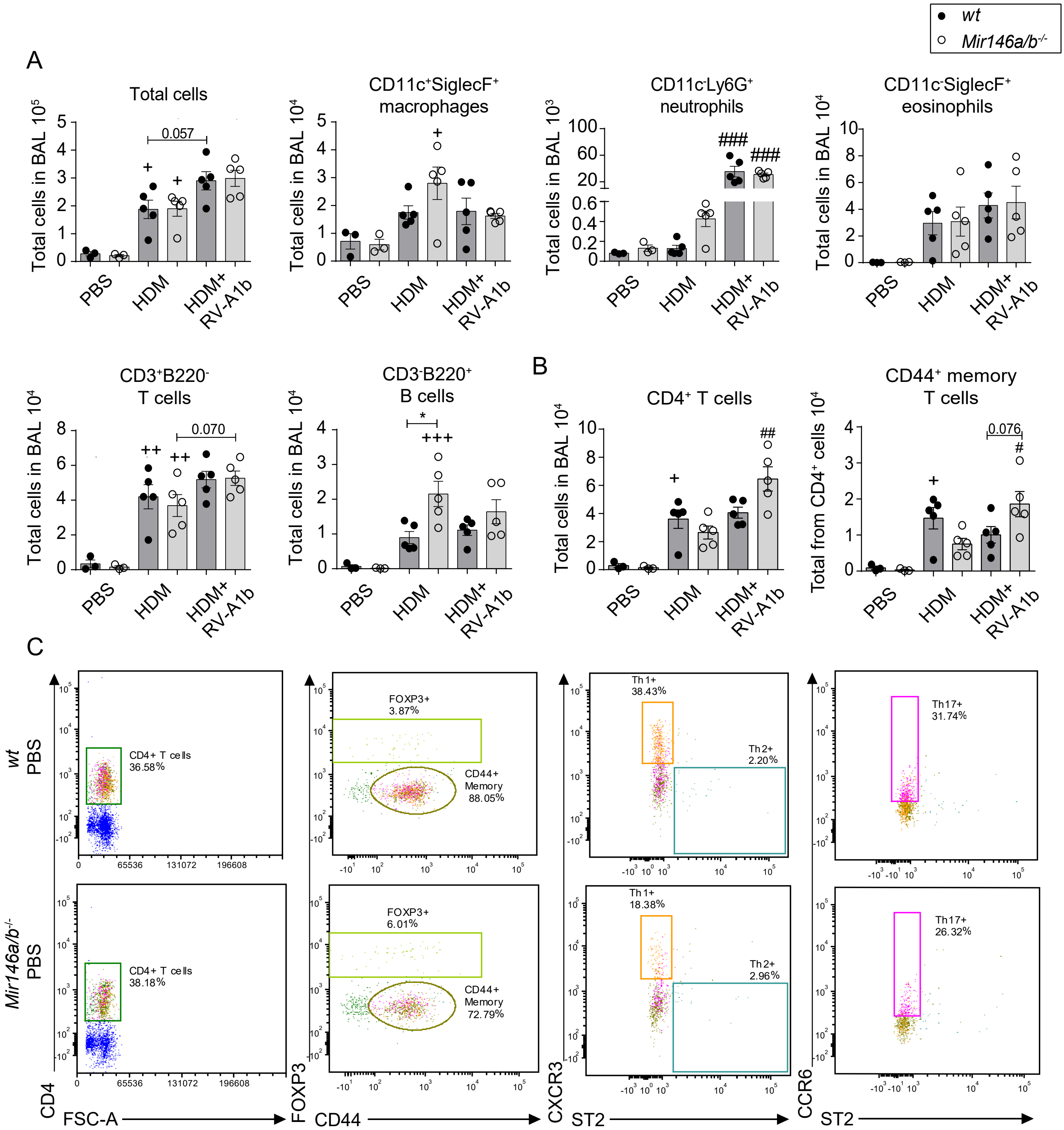


**Figure S7. Lack of miR-146a/b in mice results in an altered T cell response in allergic airway inflammation and RV-induced asthma exacerbation models.** Mice were sensitized (D0) and challenged (D7-11) with HDM extract and when indicated, infected with RV-A1b (D21) for 24h. Control mice received only PBS. (A-B) BAL cells were counted with a hemocytometer and then subjected to FACS analysis. From the dot plot percentages, the total numbers of immune cells were calculated. Data represent mean ± SEM. One-way ANOVA with Tukey's multiple comparisons test and adjusted P-values are shown. + $P < 0.05$, ++ $P < 0.01$, +++ $P < 0.001$ HDM compared to the same mouse line with PBS; # $P < 0.05$, ## $P < 0.01$, ### $P < 0.001$ HDM+RV-A1b compared to same mouse line with HDM; * $P < 0.05$ *wt* compared to *Mir146a/b*$^{-/-}$ mice for the same treatment. (C) One representative FACS dot-plot of BAL from three PBS treated *wt* and *Mir146a/b*$^{-/-}$ mice are shown.

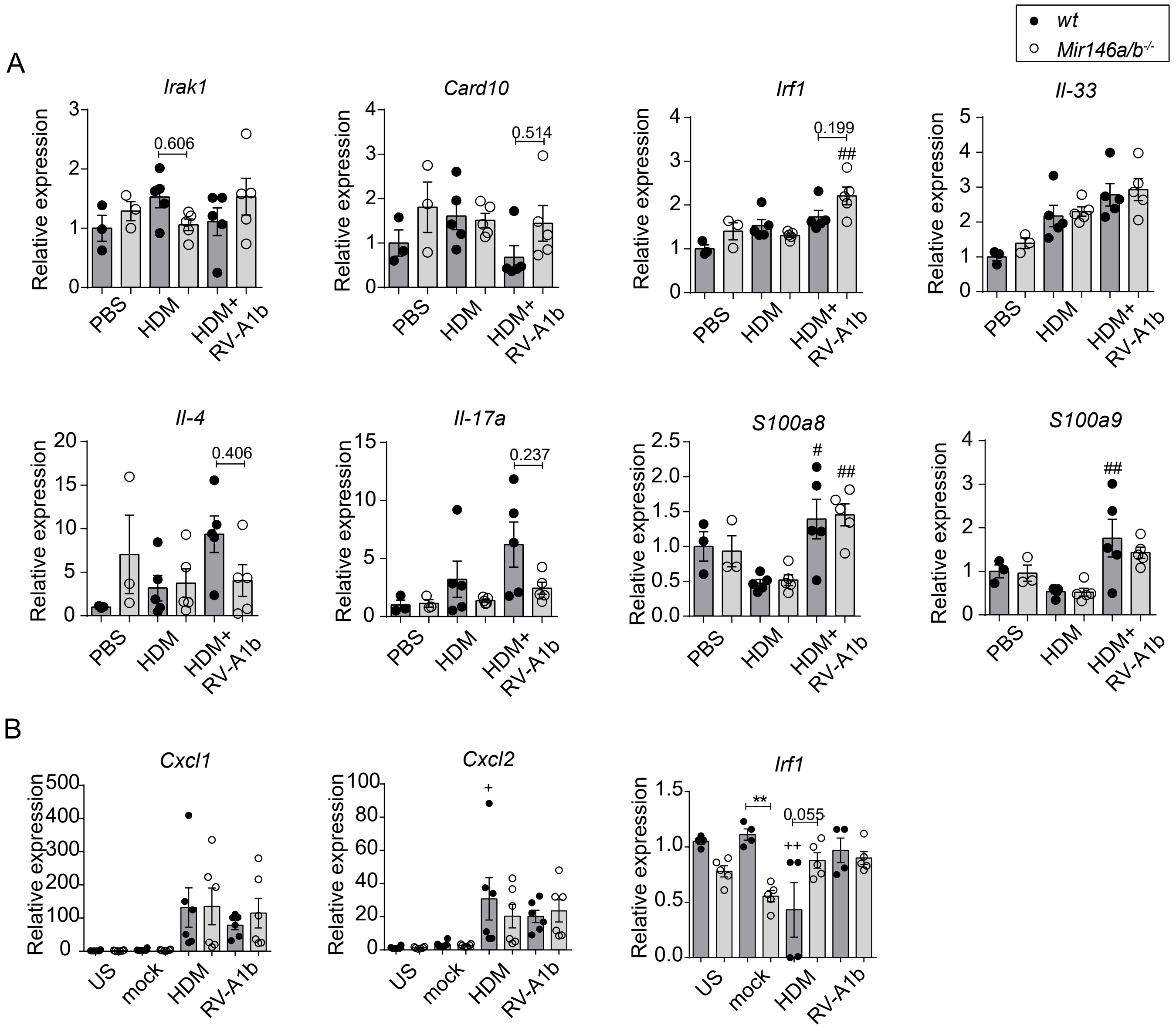


**Figure S8.** ***Mir146a/b*$^{-/-}$ mice display differential gene expression in their lungs and BMDCs in response to HDM or RV-A1b.** Relative mRNA (A-B) expression in mouse lungs subjected to HDM induced airway inflammation and RV induced asthma exacerbation models (A), in BMDCs (B) as measured by RT-qPCR. Data are presented as mean ± SEM. One-way ANOVA with Tukey's multiple comparisons test, adjusted P values are shown. (A) # $P < 0.05$, ## $P < 0.01$ HDM+RV compared to same line HDM (B) ** $P < 0.01$ *wt* compared to *Mir146a/b*$^{-/-}$ during same treatment, + $P < 0.05$, ++ $P < 0.01$ HDM compared to same mouse line US.

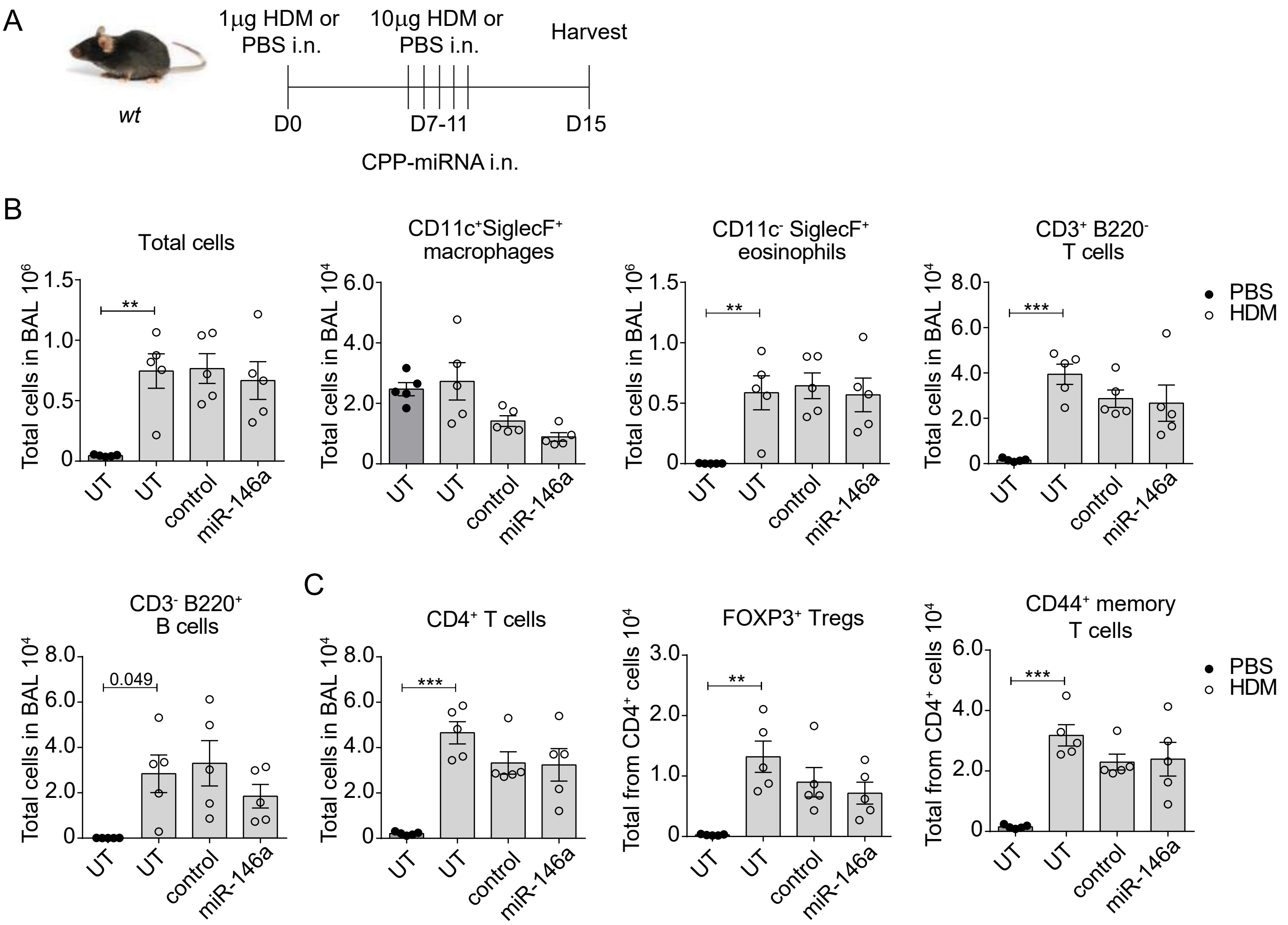


**Figure S9. The effect of intranasal application of CPP-miRNA nanocomplexes in allergic airway inflammation model.** (A) *Wt* mice were subjected to HDM-induced airway inflammation model. CPP-control or CPP-miR-146a nanocomplexes were i.n. administered 2h before each challenge. (B-C) For untransfected (UT) PBS group, only PBS and UT HDM group, only HDM was applied. BAL cells from five mice in each group were counted with a hemocytometer, subjected to flow cytometry and total numbers of immune cells were calculated from dot-plots. Data represent mean ± SEM. One-way ANOVA with Tukey's multiple comparisons test, adjusted P values are shown, ** $P < 0.01$, *** $P < 0.001$.

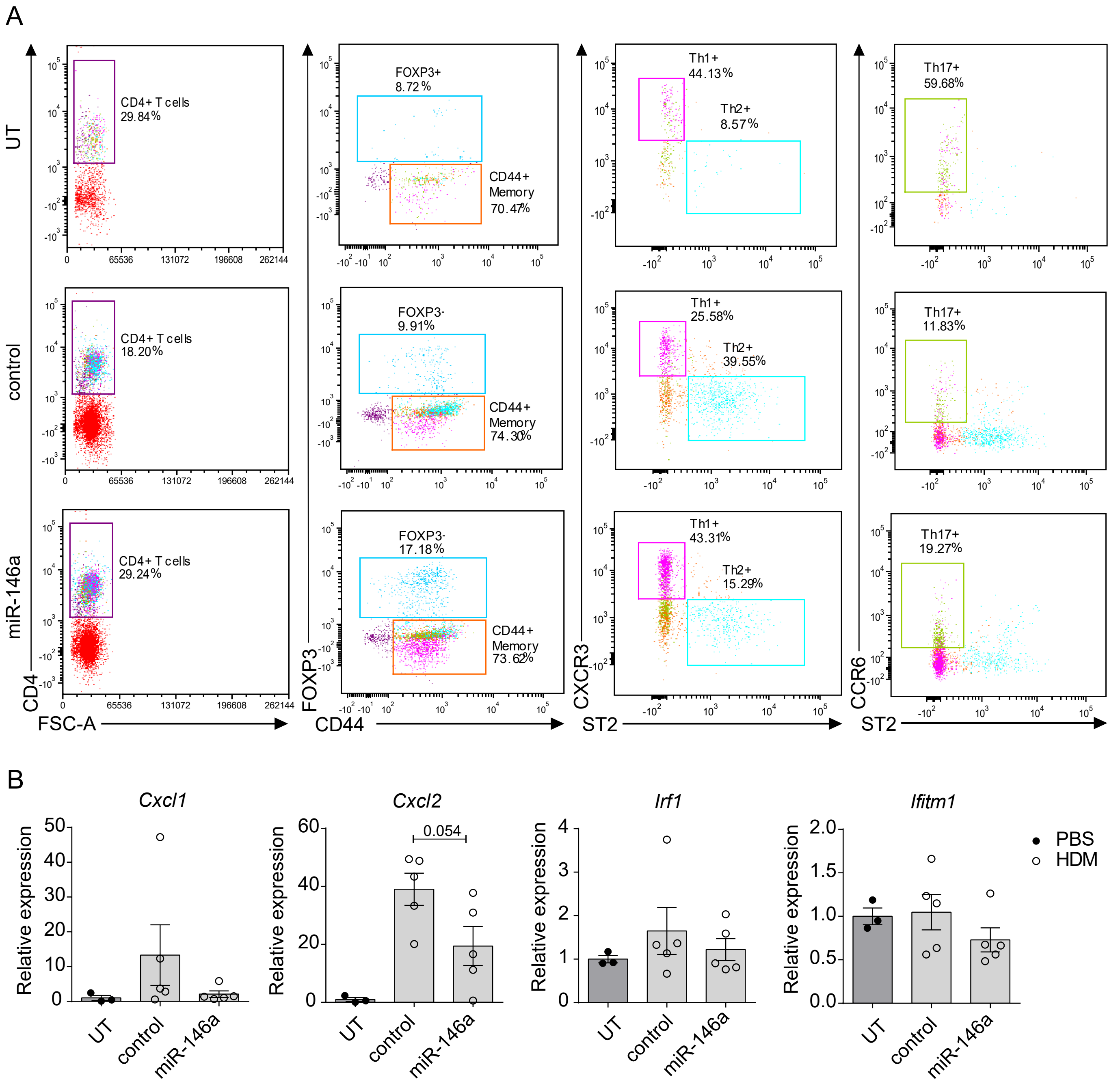


**Figure S10. The effect of CPP-miR-146a nanocomplexes on T cells and gene expression of selected pro-inflammatory cytokines and interferon response genes in allergic airway inflammation model.** *Wt* mice were subjected to HDM induced airway inflammation model. 2h after each challenge, CPP-miR-146a or CPP-control nanocomplexes were i.n. administered. Control mice received no CPP-miRNA nanocomplexes, only PBS. (A) One representative FACS dot-plot of BAL from three PBS treated, five HDM and control miRNA mimics treated, and five HDM and miR-146a treated *wt* mice. (B) Relative mRNA expression in mouse lungs as measured by RT-qPCR. Data represent mean ± SEM.
